# Circulating miR-103a-3p contributes to angiotensin II-induced renal inflammation and fibrosis via a SNRK/NF-κB/p65 regulatory axis

**DOI:** 10.1038/s41467-019-10116-0

**Published:** 2019-05-13

**Authors:** Qiulun Lu, Zejun Ma, Ye Ding, Tatiana Bedarida, Liming Chen, Zhonglin Xie, Ping Song, Ming-Hui Zou

**Affiliations:** 1 Center for Molecular and Translational Medicine, Georgia State University, Atlanta, GA USA; 2 Key Laboratory of Hormones and Development (Ministry of Health), Tianjin Key Laboratory of Metabolic Diseases, Tianjin Metabolic Diseases Hospital & Tianjin Institute of Endocrinology, Tianjin Medical University, Tianjin, China

**Keywords:** miRNAs, Renal fibrosis

## Abstract

Although angiotensin II (AngII) is known to cause renal injury and fibrosis, the underlying mechanisms remain poorly characterized. Here we show that hypertensive nephropathy (HN) patients and AngII-infused mice exhibit elevated levels of circulating miR103a-3p. We observe a positive correlation between miR-103a-3p levels and AngII-induced renal dysfunction. miR-103a-3p suppresses expression of the sucrose non-fermentable-related serine/threonine-protein kinase SNRK in glomerular endothelial cells, and glomeruli of HN patients and AngII-infused mice show reduced endothelial expression of SNRK. We find that SNRK exerts anti-inflammatory effects by interacting with activated nuclear factor-κB (NF-κB)/p65. Overall, we demonstrate that AngII increases circulating miR-103a-3p levels, which reduces SNRK levels in glomerular endothelial cells, resulting in the over-activation of NF-κB/p65 and, consequently, renal inflammation and fibrosis. Together, our work identifies miR-103a-3p/SNRK/NF-κB/p65 as a regulatory axis of AngII-induced renal inflammation and fibrosis.

## Abstract (web summary)

Angiotensin II is known to cause renal inflammation and fibrosis. Here Lu et al. show that levels of circulating miR-103a-3p are elevated in hypertensive nephropathy patients and in an animal model of angiotensin II-induced renal dysfunction, and that miR-103a-3p suppresses SNRK expression leading to the activation of the pro-inflammatory NF-κB pathway in glomerular endothelial cells.

## Introduction

The renin–angiotensin–aldosterone system (RAAS) controls renal function and arterial pressure^1^. Renin, released by the kidneys, facilitates the production of angiotensin II (AngII), which in turn stimulates the release of aldosterone from the adrenal cortex^2^. As the main effector of the RAAS, AngII can exert both a vasoconstrictor effect and a pro-inflammatory action on post-glomerular arteries, resulting in vascular and glomerular injuries and consequently necrosis and fibrosis of the kidneys^3–6^. This renal damage manifests as elevated glomerular protein filtration, extracellular matrix deposition, glomerular fibrosis, and inflammation^7–9^. Although increasing evidence indicates that pathological doses of AngII are responsible for aberrant inflammation and fibrosis in renal diseases^10,11^, the underlying molecular mechanisms remain poorly understood.

MicroRNAs (miRNAs) are small, non-coding RNAs that bind to the complementary sequences of messenger (m)RNAs to inhibit mRNA translation or to promote mRNA degradation^12^. These RNA species are usually stable and have long half-lives in biological fluids. Renal physiology and pathology are associated with three types of miRNAs: urinary miRNAs, circulating miRNAs, and kidney miRNAs^13,14^. Elevated levels of some circulating miRNAs, including miR-144-5p and miR-1825, positively correlate with the severity of renal injury^15,16^. Reduced levels of other circulating miRNAs, including miR-30d, miR-140-3p, miR-532-3p, and miR-190, have been observed in patients with end-stage renal disease^17,18^. Various circulating miRNAs present in plasma function as mediators of cell-to-cell communication and play important roles in renal pathogenesis associated with various disorders, including cardiovascular diseases^19–21^, diabetes^22^, and cancer^23–25^. Despite these strong associations between circulating miRNAs and kidney diseases, the causal relationship between circulating miRNAs and renal dysfunction remains unclear.

Plasma of hypertension patients contains elevated levels of miR-103 relative to that of healthy individuals, as revealed by miRNA microarray analysis^26^. Similarly, urine of diabetes mellitus patients contains higher miR-103 levels than that of healthy controls^27^. Overexpression of miR-103 aggravates endoplasmic reticulum (ER)-mediated apoptosis in preadipocytes via WNT family member 3a (Wnt3a) regulation^28^. Conversely, in human umbilical vein endothelial cells (HUVECs), miR-103 suppresses the accumulation of H_2_O_2_-induced reactive oxygen species by reducing Bcl2/adenovirus E1B 19 kDa protein-interacting protein 3 (BNIP3) levels^29^. However, whether and how miR-103 causes renal dysfunction remains unknown.

The present study aimed to elucidate the underlying miRNA-dependent mechanisms by which AngII induces renal inflammation and injury, as well as to assess a potential link to miR-103a-3p. We present evidence that an AngII-induced increase in circulating miR-103a-3p suppresses the expression of the sucrose non-fermentable-related serine/threonine-protein kinase (SNRK) in renal endothelial cells, thereby potentiating nuclear-factor kappa B (NF-κB)/p65 signaling and promoting inflammation and injury in the kidneys.

## Results

### miR-103a-3p is upregulated in HN patients

**Fig. 1:**
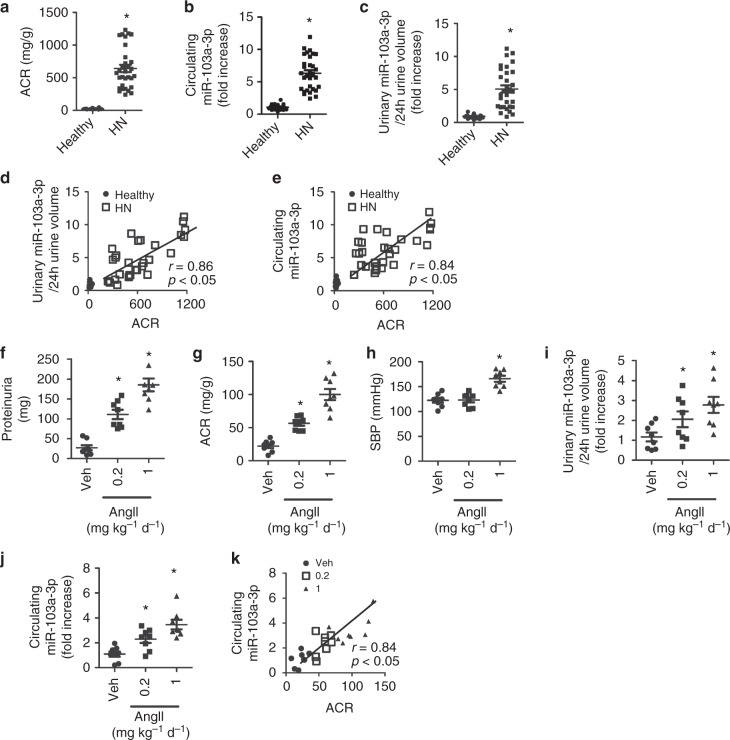
Circulating miR-103a-3p is upregulated in hypertensive nephropathy patients and AngII-treated mice. **a**–**c** Albumin/creatinine ratio (ACR) (**a**), serum miR-103a-3p levels (**b**), and ratio for urinary miR-103a-3p levels to the volume of 24 h urine (**c**) of hypertensive nephropathy (HN) patients (*n* = 31) and healthy controls (*n* = 18). *p *<* 0.05 relative to healthy controls (Student’s *t*-test). **d**, **e** Correlation between ACR and the ratio for urinary miR-103a-3p levels to the volume of 24 h urine (**d**) or serum miR-103a-3p levels (**e**) in healthy individuals and HN patients. **f**–**k** Albumin excretion rates (**f**), ACR (**g**), systolic blood pressure (SBP) (**h**), ratio for urinary miR-103a-3p levels to the volume of 24 h urine (**i**), and serum miR-103a-3p levels (**j**) in wild-type mice (*n* = 8) infused with vehicle (Veh) or AngII (0.2 or 1 mg kg^−1^ d^−1^) for 4 weeks. Urinary and serum miR-103a-3p levels were analyzed by qRT-PCR. *p < 0.05, relative to vehicle control. Statistical analysis was carried out with one-way analysis of variance. **k** Correlation between circulating miR-103a-3p levels and ACR in vehicle- or AngII-infused mice. Data are mean ± SEM. Each symbol represents an individual sample. The corresponding source data are available in the Source Data file

To determine the relationship between circulating microRNAs and AngII-induced renal injury, we examined miR-103a-3p levels in human urine and blood samples from normotensive healthy controls (*n* = 18) and patients with hypertensive nephropathy (HN, *n* = 31), detected using a blood pressure threshold of >130/90 mm Hg and renal pathology (Supplementary Table 1). The albumin/creatinine ratio (ACR), an indicator of renal function, was markedly elevated in HN patients relative to controls (Fig. 1a). Serum and urine levels of miR-103a-3p were also significantly higher in HN patients than in healthy subjects (Fig. 1b and Supplementary Fig. 1a). No urine volume changes were detected, as similar trends were observed when urinary miR-133a-3p levels were adjusted to 24-h urine volume (Fig. 1c). The ACR was positively correlated with miR-103a-3p levels in both urine and serum samples (Fig. 1d, e, and Supplementary Fig. 1b). Both Diovan (β-blockers) and Monopril (ACEi) induced the reduction of urine and serum levels of miR-103a-3p only in HN patients with reduced ACR (Supplementary Fig. 1c–h).

### Expression of miR-103a-3p increases in AngII-infused mice

We reasoned that either arterial pressure or AngII increases circulating miRNA levels in HN patients. To differentiate a direct effect of AngII and AngII-mediated high blood pressure, we administered mice with one of two AngII doses (0.2 and 1 mg kg^−1^ d^−1^) or vehicle control for 4 weeks. Infusion with the low dose of AngII (0.2 mg kg^−1^ d^−1^) caused proteinuria (Fig. 1f) and increased the ACR (Fig. 1g), without altering systolic blood pressure (SBP) (Fig. 1h), indicating that low-dose AngII caused blood pressure-independent renal injury. However, the high dose of AngII induced both renal injury and hypertension (Fig. 1f, h). Either dose of AngII significantly increased urine and serum levels of miR-103a-3p relative to the vehicle control, with the most substantial increases observed following administration of the higher dose (Fig. 1i, j and Supplementary Fig. 1i). Like the human subjects, the mice also demonstrated a positive correlation between serum miR-103a-3p levels and ACR (Fig. 1k). These data indicate that AngII can increase miR-103a-3p expression independently of arterial pressure.

### miR-103a-3p elevation aggravates AngII-induced renal injury

**Fig. 2:**
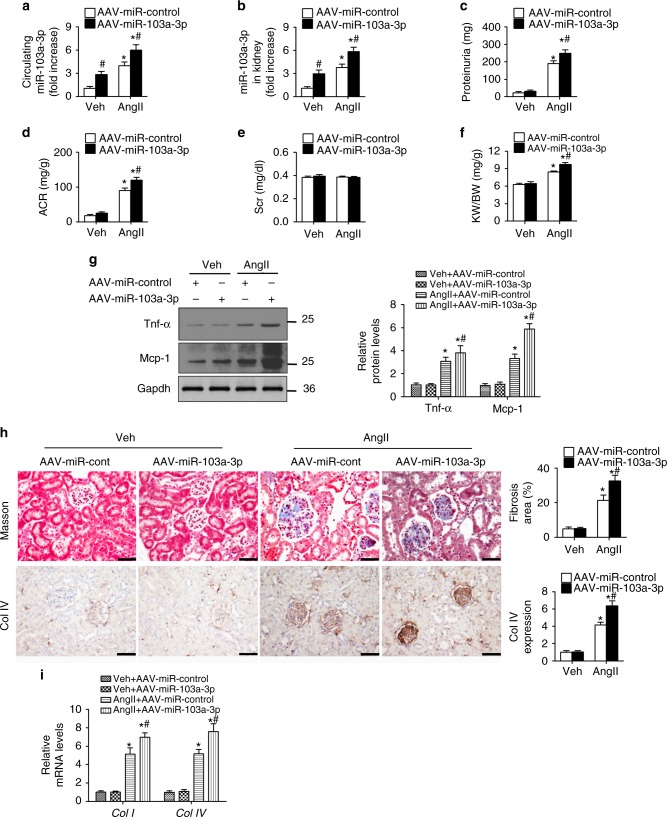
Overexpression of miR-103a-3p promotes AngII-induced renal injury. Mice (*n* = 6 per group) transfected with AAV-miR-103a-3p or AAV-miR-control were infused with a vehicle control (Veh) or AngII (1 mg kg^−1^ d^−1^) for 4 weeks. **a** Serum miR-103a-3p levels as determined by qRT-PCR. **b** Kidney tissue miR-103a-3p levels. **c** Albumin excretion rate. **d** Albumin/creatinine ratio (ACR). **e** Serum creatinine levels. **f** Kidney weight to body weight ratio (KW/BW). **g** Western blot analysis of Mcp-1 and Tnf-α levels in the kidneys. **h** Representative images of trichrome staining and immunohistochemical staining of collagen type IV (Col IV) in the kidneys (scale bar = 50 µm). **i** Collagen type I and IV mRNA levels as measured using qRT-PCR. For all experiments, *n* = 6 per group; *p < 0.05 relative to vehicle control; ^#^*p* < 0.05 relative to AAV-miR-Control (Student’s *t*-test). The corresponding source data are available in the Source Data file

To evaluate the role of miR-103a-3p in AngII-induced renal injury, we overexpressed miR-103a-3p in mice using a recombinant adeno-associated viral (AAV) system. Delivery of this AAV system significantly increased serum and kidney miR-103a-3p levels (Fig. 2a, b). As expected, miR-103a-3p overexpression significantly increased urinary albumin, ACR, and the ratio of kidney weight to body weight in AngII-infused mice, while there is no significant change for serum creatinine levels (Fig. 2c–f). Upregulation of monocyte chemoattractant protein-1 (Mcp-1), tumor necrosis factor (Tnf)-α, interleukin (Il)-6, and Il-18 has been previously observed in the kidney tissues of AngII-infused mice^5,6^. Presently, miR-103a-3p overexpression elevated *Mcp-1*, *Tnf-α*, *Il-6*, and *Il-18* mRNA levels and increased Mcp-1 and Tnf-α protein levels in AngII-infused mice (Fig. 2g and Supplementary Fig. 2). Overexpression of miR-103a-3p also aggravated the AngII-induced inflammatory response in vivo, as evidenced by intensified trichrome staining and increased mRNA and protein levels of collagen type I and IV (Fig. 2h, i). These findings suggest that increased expression of miR-103a-3p further enhances AngII-induced renal inflammation and injury.

### Loss of miR-103a-3p ameliorates AngII-induced renal injury

**Fig. 3:**
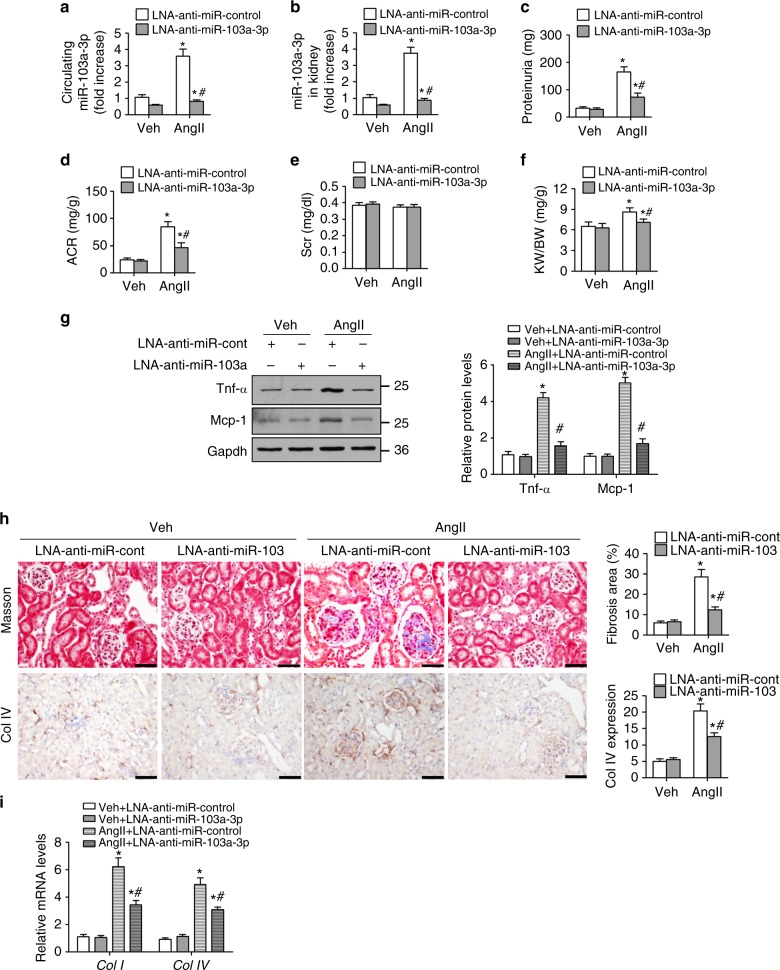
Reduction of miR-103a-3p ameliorates AngII‐induced renal injury. Mice (*n* = 8 per group) injected with LNA-anti-miR-103a-3p or LNA-anti-miR-control were infused with a vehicle control (Veh) or AngII (1 mg kg^−1^ d^−1^) for 4 weeks. **a** Serum miR-103a-3p levels were analyzed by qRT-PCR. **b** Kidney tissue miR-103a-3p levels. **c** Albumin excretion rate. **d** Albumin/creatinine ratio (ACR). **e** Serum creatinine levels. **f** Kidney weight to body weight ratio (KW/BW). **g** Western blot analysis of Mcp-1 and Tnf-α expression in the kidneys. **h** Representative images of trichrome staining and immunohistochemical staining of collagen type IV (Col IV) in the kidneys (scale bar = 50 µm). **i** Collagen type I and IV mRNA levels were measured using qRT-PCR. For all experiments, *n* = 8 per group; *p < 0.05 relative to vehicle control; ^#^*p* < 0.05 relative to LNA-anti-miR-Control. Statistical analysis was carried out by a Student’s two-tailed *t*-test. The corresponding source data are available in the Source Data file

We next asked whether loss of miR-103a-3p can alleviate AngII-induced renal injury. Knockdown of miR-103a-3p using locked nucleic acid (LNA)-anti-miR-103a-3p suppressed AngII-induced elevation of miR-103a-3p in blood and kidney tissue (Fig. 3a, b). The knockdown also eliminated proteinuria and increased ACR and kidney weight to body weight ratios in the AngII-treated mice (Fig. 3c–f). LNA-anti-miR-103a-3p administration also decreased *Mcp-1*, *Tnf-α*, *Il-6*, and *Il-18* mRNA levels and Mcp-1 and Tnf-α protein abundance (Fig. 3g and Supplementary Fig. 3), indicating suppression of AngII-induced inflammation. The LNA-anti-miR-103a-3p-treated mice also exhibited reduced AngII-induced renal fibrosis, as evidenced by decreased trichrome staining and reduced expression of collagen type I and IV (Fig. 3h, i). Similar reductions in miR-103a-3p levels (Supplementary Fig. 4a and b), renal injury (Supplementary Fig. 4c–f), inflammation (Supplementary Fig. 5a and b), and renal fibrosis (Supplementary Fig. 5c and d) were observed following knockdown of miR-103a-3p using AAV-anti-miR-103a-3p. Taken together, these findings suggest that loss or inhibition of miR-103a-3p can reduce AngII-induced renal inflammation and injury.

### SNRK is a target of miR-103a-3p

**Fig. 4:**
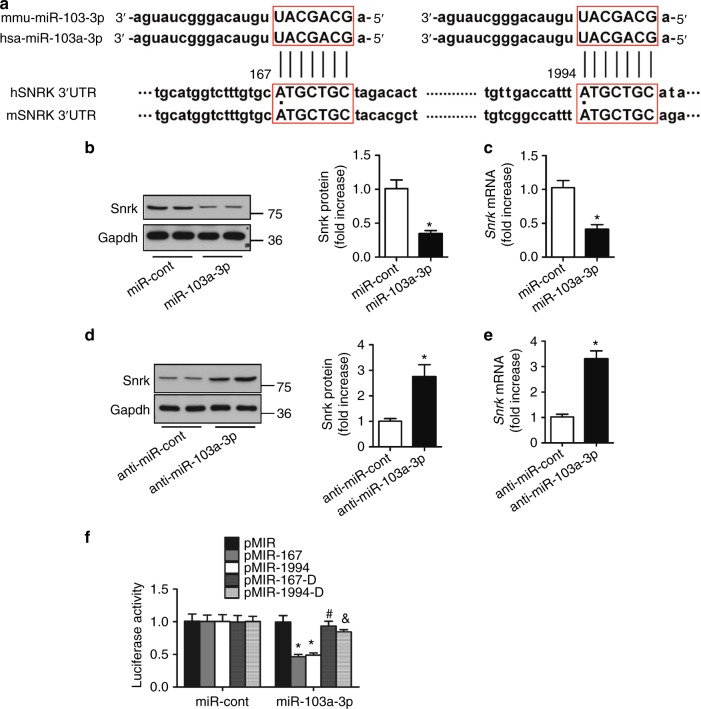
SNRK is a target of miR-103a-3p. **a** Sequence analysis of human and mouse miR-103a-3p and *SNRK* mRNA 3′UTR. Putative miR-103a-3p and *SNRK* mRNA 3′UTR binding regions are highlighted with red boxes. **b**–**e** Primary GnECs were transfected with miR-103a-3p (**b**, **c**) or anti-miR-103a-3p (**d** and **e**) for 36 h. Snrk protein levels were assessed using Western blotting and densitometry (**b**, **d**), and *Snrk* mRNA abundance was measured using qRT-PCR (**c**, **e**). For all experiments, *n* = 6 per group; *p < 0.05 relative to miR-Control. **f** Luciferase activity assays were performed to assess whether the regulatory effect of miR-103a-3p requires the predicted binding sites in the *SNRK* mRNA 3′UTR in HEK293 cells. pMIR167 and pMIR1994 harbored the 167 and 1994 putative binding sites, respectively (indicated in **a**), whereas the respective binding sites were individually deleted in pMIR167∆ and pMIR1994∆. *n* = 6 per group, *p < 0.05 relative to pMIR; ^#^*p* < 0.05 relative to pMIR-167; ^&^*p* < 0.05 relative to pMIR-1994. Statistical analysis was carried out with a Student’s two-tailed *t*-test. The corresponding source data are available in the Source Data file

To predict the effective target sites of miR-103a-3p, which was upregulated in both hypertensive patients and AngII-infused mice, we performed computational target-scan analysis^30^. The analysis identified two highly conserved miR-103a-3p binding sites within the *SNRK* mRNA 3′ untranslated region (3′UTR) at positions 167–173 and 1994–2000 (Fig. 4a). To assess whether miR-103a-3p regulates SNRK expression, we examined Snrk protein and mRNA levels in glomerular endothelial cells (GnECs) transfected with miR-103a-3p or anti-miR-103a-3p. Overexpression of miR-103a-3p significantly reduced Snrk expression (Fig. 4b, c), whereas anti-miR-103a-3p increased mRNA and protein levels of Snrk (Fig. 4d, e).′

Next, we used a luciferase reporter assay to test whether the regulatory effect of miR-103a-3p requires the predicted binding sites in the *SNRK* 3′UTR. Overexpression of miR-103a-3p resulted in a significant decrease in luciferase activity in human embryonic kidney (HEK) 293 cells transfected with pMIR167 and pMIR1994, harboring the 167 and 1994 binding sites, respectively (Fig. 4f). However, this effect was abolished in cells transfected with either pMIR167∆ or pMIR1994∆, in which the predicted 167 and 1994 binding sites were individually deleted (Fig. 4f), suggesting that miR-103a-3p binds to the 3′UTR of *SNRK* to inhibit its expression.

The effect of miR-103a-3p was confirmed in vivo, as AAV-miR-103a-3p downregulated *Snrk* expression in mouse kidney tissues (Supplementary Fig. 6a and b). Conversely, in vivo miR-103a-3p knockdown using AAV-anti-miR-103a-3p increased Snrk levels in kidney tissues (Supplementary Fig. 6c and d), while reducing AngII-induced renal injury (Supplementary Fig. 5c and d).

### Renal *SNRK* expression is downregulated in HN patients

**Fig. 5:**
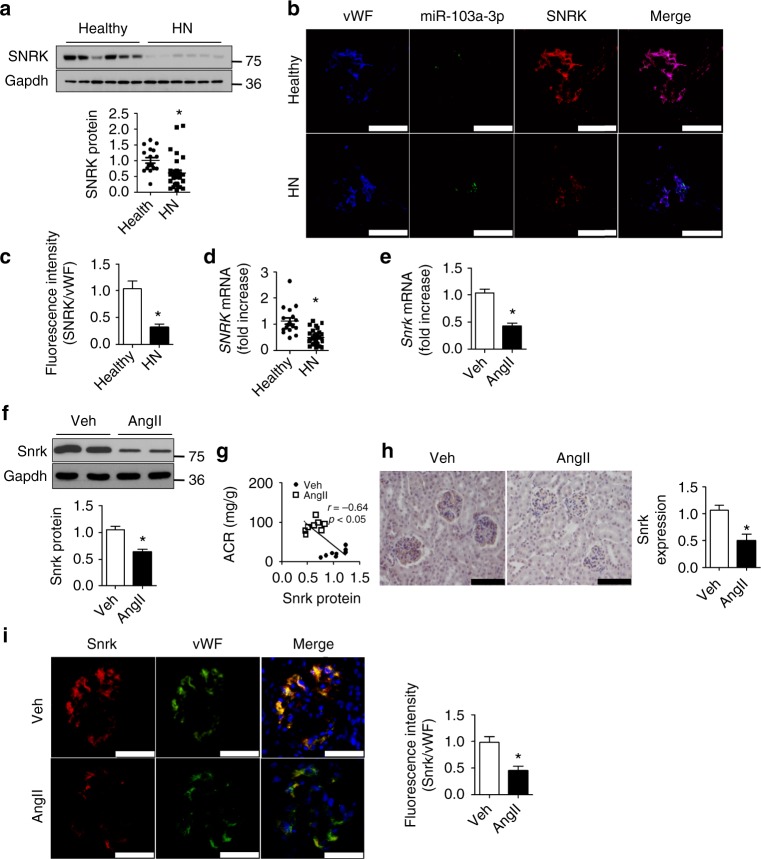
SNRK protein is downregulated in renal endothelium of hypertensive nephropathy patients and AngII-treated mice. **a** SNRK protein levels in kidney tissues of healthy individuals and hypertensive nephropathy (HN) patients. *p *<* 0.05 relative to healthy controls. **b** Immunofluorescent co-staining of miR-103a-3p (green), SNRK (red), and Von Willebrand factor (vWF; blue) in kidney tissues of healthy controls and HN patients (scale bar = 50 µm). **c** Quantitative analysis of the colocalization of SNRK and vWF immunofluorescence signals in kidney tissues. *n* = 6; *p *<* 0.05 relative to healthy controls. **d** *SNRK* mRNA levels in kidney tissues were measured by qRT-PCR. *n* = 6; *p *<* 0.05 relative to healthy controls. **e**-**i** Mice were infused with a vehicle control (Veh) or AngII (1 mg kg^−1^ d^−1^) for 4 weeks. **e** *Snrk* mRNA levels in kidney tissues were measured using qRT-PCR. **f** Snrk protein levels in kidney tissues were measured using Western blot analysis. **g** Correlation between renal Snrk protein levels and the albumin/creatinine ratio in mice with or without AngII infusion. **h** Immunohistochemical staining of Snrk in mouse kidney sections (scale bar = 100 µm). **i** Immunofluorescence staining of Snrk (red) and vWF (green) in kidney sections from vehicle- or AngII-treated mice (scale bar = 50 µm). For murine experiments, *n* = 8; *p *<* 0.05 relative to vehicle control. Statistical analysis was carried out with a Student’s two-tailed *t*-test. The corresponding source data are available in the Source Data file

To establish the relationship between SNRK and HN, we first examined *SNRK* expression in micro-dissected human kidney samples from patients with HN and control normotensive individuals. SNRK protein levels were significantly lower in HN kidneys than in control kidneys (Fig. 5a). Next, we investigated the cellular distribution of SNRK in the kidneys. Immunofluorescence microscopy revealed that SNRK staining colocalized with Von Willebrand factor (vWF)-positive cells, suggesting that SNRK is mainly expressed in renal endothelial cells (Fig. 5b). Fluorescence in situ hybridization (FISH) indicated that miR-103a-3p also colocalized with vWF-positive cells (Fig. 5b and Supplementary Fig. 7). The fluorescence intensity ratios for SNRK to vWF signals were substantially lower in GnECs from HN patients than in those from the healthy cohort (Fig. 5c). Accordingly, quantitative real-time polymerase chain reaction (qRT-PCR) assays showed a reduction in *SNRK* mRNA levels in kidney tissues from HN patients relative to those from the healthy controls (Fig. 5d).

### AngII infusion leads to *Snrk* reduction and renal injury

Considering the effect of AngII on miR-103a-3p and of miR-103a-3p on SNRK, we next tested whether AngII infusion alters *Snrk* expression in mice in vivo^5,31^. AngII infusion and the AngII-induced renal injury were associated with significant reductions in Snrk mRNA and protein levels in the kidneys (Fig. 5e, f). We found an inverse relationship between Snrk protein levels and ACR (Fig. 5g), with Snrk mainly detected in the glomeruli (Fig. 5h). As observed in the human kidney samples, Snrk colocalized with vWF, a specific GnEC marker, in murine kidneys (Fig. 5i). Collectively, these data suggest that AngII lowers Snrk levels in mouse renal endothelial cells.

We next assessed whether the AngII effects on Snrk are miR-103a-3p-dependent. Like human subjects, mice demonstrated a negative correlation between urine and serum miR-103a-3p levels and Snrk expression (Supplementary Fig. 8a and b). Furthermore, FISH staining of kidney tissues from AngII-infused mice indicated that AAV-miR-103a-3p administration increased the expression of miR-103a-3p and reduced Snrk levels in endothelial cells (Supplementary Fig. 9).

### Serum from AngII-infused mice reduces *Snrk* in cultured GnECs

**Fig. 6:**
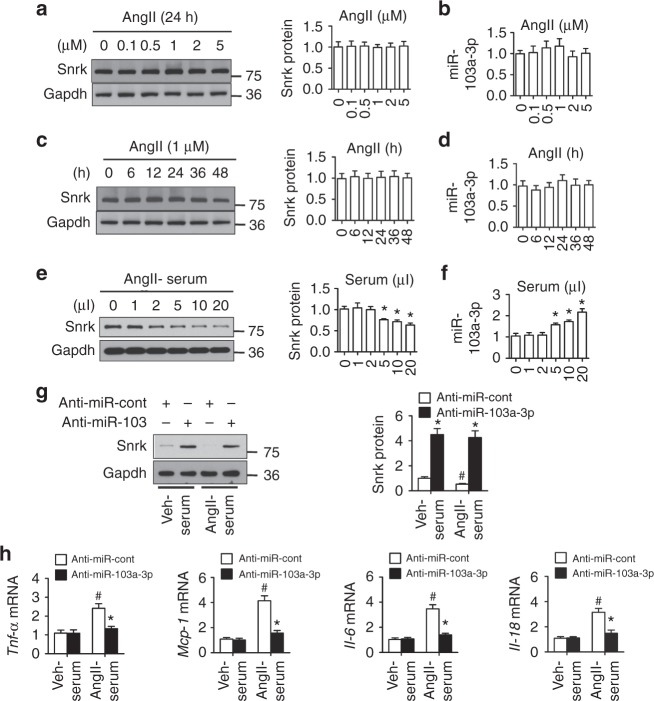
AngII-infused mouse serum reduces Snrk expression in cultured GnECs. **a** Western blot and densitometry analysis of Snrk levels in primary GnECs isolated from wild-type (WT) mice following treatment with different doses of AngII. **b** Quantification of miR-103a-3p levels by qRT-PCR in primary GnECs treated with different doses of AngII. **c** Western blot and densitometry analysis of Snrk levels in GnECs isolated from WT mice following different incubation times with 1 µM AngII. **d** Quantification of miR-103a-3p levels by qRT-PCR in primary GnECs treated with AngII for different durations. e–h Serum collected from WT mice infused with a vehicle control (Veh) or AngII (1 mg kg^−1^ d^−1^) for 4 weeks was used to treat GnECs. Snrk proteins levels (**e**) and miR-103a-3p abundance (**f**) were determined for GnECs treated for 8 h with different doses of AngII-treated mouse serum. *n* = 6; *p < 0.05 relative to untreated controls. GnECs pretreated with AAV-anti-miR-103a-3p or AAV-anti-miR-Control for 24 h and subsequently incubated with vehicle control- or AngII-treated mouse serum (10 µl) were subjected to the analysis of Snrk protein levels (**g**) and cytokine mRNA levels (**h**). *n* = 6 per group; *p < 0.05 relative to anti-miR-Control/AngII; ^#^*p* < 0.05 relative to vehicle-treated mouse serum. Student’s two-tailed *t*-test was used for the statistical analysis. The corresponding source data are available in the Source Data file

When tested in vitro, AngII alone did not alter Snrk expression in GnECs at doses up to 5 μM (Fig. 6a and Supplementary Fig. 10a). Similarly, high-dose AngII did not affect miR-103a-3p levels (Fig. 6b). Prolonged incubation with AngII for 48 h also did not alter Snrk or miR-103a-3p levels in GnECs (Fig. 6c, d, and Supplementary Fig. 10b). These results suggest that AngII-induced elevation of circulating miR-103a-3p likely causes Snrk reduction in AngII-infused mice in vivo.

Thus, we tested whether serum collected from AngII-infused mice could modulate Snrk levels in GnECs. Interestingly, serum from AngII-infused mice suppressed Snrk expression at the mRNA and protein levels in a dose-dependent manner (Fig. 6e and Supplementary Fig. 10c), and increased miR-103a-3p levels in GnECs (Fig. 6f). To assess whether this effect was dependent on circulating miR-103a-3p, we pretreated the GnECs with AAV-anti-miR-103a-3p or AAV-anti-miR-Control before exposure to serum from vehicle- or AngII-treated mice. Knockdown of miR-103a-3p abolished the suppression on Snrk expression in GnECs caused by AngII-infused mouse serum (Fig. 6g and Supplementary Fig. 10d), suggesting that Snrk levels in GnECs were regulated specifically by circulating miR-103a-3p from AngII-infused mice. Furthermore, miR-103a-3p silencing in GnECs suppressed the inflammatory response to the serum of AngII-infused mice, as evidenced by reduced cytokine mRNA levels (Fig. 6h).

### EC *Snrk* deletion exacerbates AngII-induced renal injury

**Fig. 7:**
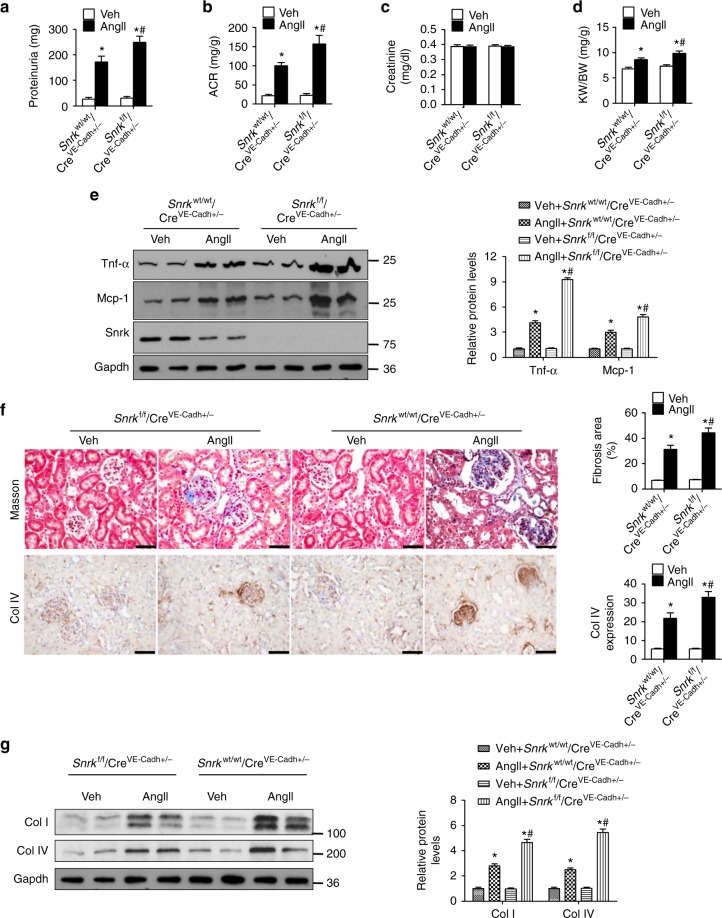
Deletion of endothelial *Snrk* exacerbates AngII-induced renal injury. Eight-week-old male wild-type (WT, *Snrk*^wt/wt^/Cre^VE-Cadh+/−^) and *Snrk* endothelial-specific knockout (*Snrk*^f/f^/Cre^VE-Cadh+/−^) mice were infused with a vehicle control or AngII for 4 weeks. **a** Albumin excretion rates. **b** Albumin/creatinine ratio. **c** Serum creatinine levels. **d** Kidney weight to body weight ratio. **e** Western blot analysis of Mcp-1 and Tnf-α levels in mouse kidneys. **f** Representative images of trichrome staining and immunohistochemical staining of collagen type IV (Col IV) in kidney tissues (scale bar = 50 µm). **g** Western blot analysis of collagen type I and IV levels in mouse kidneys. For all experiments, *n* = 8 per group; *p < 0.05 relative to vehicle control; ^#^*p* < 0.05 relative to WT mice/AngII. Statistical analysis was carried out with a Student’s two-tailed *t*-test. The corresponding source data are available in the Source Data file

To establish the role of Snrk in the development of AngII-induced renal injury, endothelial-specific *Snrk* knockout (*Snrk*^f/f^/Cre^VE-Cadh+/−^) and their littermate wild-type (WT, *Snrk*^wt/wt^/Cre^VE-Cadh+/−^) mice were infused with AngII. Deletion of *Snrk* did not alter the SBP or the diastolic blood pressure (DBP) (Supplementary Fig. 11a and b). AngII infusion increased SBP in both *Snrk*^*f/f*^*/Cre*^*VE-Cadh+/−*^ and WT mice to a similar extent (Supplementary Fig. 11a). Deletion of endothelial-cell *Snrk* did not impair kidney structure or function (Fig. 7a–d). Although AngII infusion did not affect serum creatinine levels (Fig. 7c), it did increase proteinuria, ACR, and kidney-to-body-weight ratios. These increases were more pronounced in *Snrk*^f/f^/Cre^VE-Cadh+/−^ relative to WT mice (Fig. 7a–d), suggesting that endothelial-specific *Snrk* deletion likely exacerbates AngII-induced renal lesions. Upon AngII exposure, *Snrk*^f/f^/Cre^VE-Cadh+/−^ mice exhibited significantly higher cytokine mRNA and protein levels relative to the WT mice (Fig. 7e and Supplementary Fig. 12a). AngII exposure similarly resulted in higher levels of renal fibrosis in *Snrk*^f/f^/Cre^VE-Cadh+/−^ mice relative to the WT mice, as evidenced by trichrome staining and collagen type I and IV expression (Fig. 7f, g, and Supplementary Fig. 12b).

### NF-κB/p65 mediates *Snrk* deletion-aggravated renal injury

**Fig. 8:**
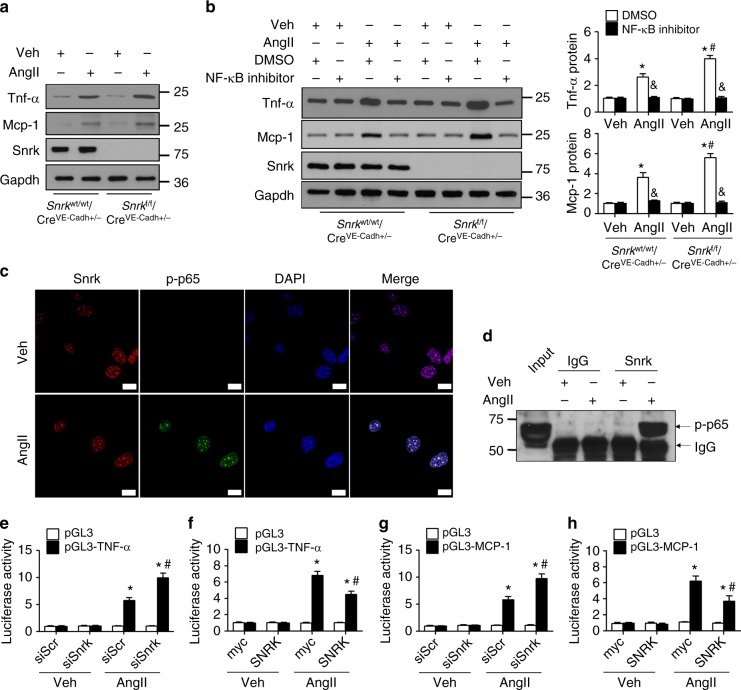
Lack of endothelial *Snrk* enhances AngII-induced inflammation by potentiating the NF-κB pathway. Primary GnECs were isolated from *Snrk*^wt/wt^/Cre^VE-Cadh+/−^ (WT) and *Snrk*^f/f^/Cre^VE-Cadh+/−^ mice. **a** Cell lysates of GnECs treated with AngII (1 µM) or a vehicle control (Veh) for 24 h were subjected to western blot analysis of Mcp-1, Tnf-α, Snrk, and Gapdh levels. **b** GnECs were pretreated with DMSO or an NF-κB inhibitor for 2 h, followed by incubation with AngII or a vehicle control for 24 h. Levels of indicated proteins were determined using western blot and densitometry analysis. *n* = 6, *p < 0.05 relative to vehicle control; ^#^*p* < 0.05 relative to GnECs from WT mice; ^&^*p* < 0.05 relative to DMSO control/AngII. **c** WT GnECs were treated with AngII for 24 h. The subcellular distribution of Snrk (red) and phosphorylated p-65 (p-p65; green) was determined by double staining (scale bar = 10 µm). **d** The interaction between SNRK and p-p65 in GnECs in the absence or presence of AngII was assessed using immunoprecipitation and western blot analysis. Input, total cell lysates; IgG, immunoprecipitated with IgG. **e**–**h** The effects of *Snrk* knockdown (**e**, **g**) or overexpression (**f**, **h**) on luciferase reporter activity from the *Tnf-α* (**e**, **f**) or the *Mcp-1* (**g**, **h**) promoter. siScr, knockdown control; siSNRK, *Snrk* knockdown; myc, Myc overexpression vector control; SNRK, *Snrk* overexpression; pGL3, empty luciferase reporter vector; pGL3-TNF-α, *Tnf* luciferase reporter; pGL3-MCP-1, *Mcp-1* luciferase reporter. **e**–**h**, *n* = 5. (**e**, **g**) *p < 0.05 relative to pGL3; ^#^*p* < 0.05 relative to si*Scr*. (**f**, **h**) *p < 0.05 relative to pGL3; ^#^*p* < 0.05 relative to myc. Student’s two-tailed *t*-test was used for the statistical analysis. The corresponding source data are available in the Source Data file

We next sought to determine how *Snrk* deficiency exacerbates AngII-induced renal injury. Considering that nuclear-factor kappa light-chain enhancer of activated B cells (NF-κB) plays a critical role in the genesis of renal damage caused by hypertension^32^, we used in vitro GnEC assays to determine whether it is involved in the inflammatory response resulting from *Snrk* deficiency. Consistent with the in vivo findings, AngII treatment increased the expression of Mcp-1 and Tnf-α at the protein and mRNA levels in GnECs from WT mice (Fig. 8a, Supplementary Fig. 13a and b). Inhibition of NF-κB/p65 using NF-κB inhibitor abrogated the enhanced expression of Mcp-1 and Tnf-α in GnECs from *Snrk*^f/f^/Cre^VE-Cadh+/−^ mice (Fig. 8b and Supplementary Fig. 14). Next, we analyzed the subcellular localization of Snrk and phosphorylated p65 in GnECs using immunofluorescence. AngII increased p65 phosphorylation and nuclear translocation (Fig. 8c). Phosphorylated p65 (p-p65) staining overlapped with Snrk staining in the nuclei of AngII-treated GnECs (Fig. 8c). Also, immunoprecipitation of Snrk followed by probing for p-p65, and vice versa, indicating that Snrk is physically associated with p-p65 (Fig. 8d and Supplementary Fig. 15), suggesting that Snrk modulates the AngII-activated NF-κB/p65 pathway that contributes to kidney damage.

We also investigated whether Snrk affects AngII-induced pro-inflammatory gene expression using luciferase activity assays. As expected, AngII increased luciferase activities from pGL3-TNF-α and pGL3-MCP-1 in GnECs transfected with control siRNA or Myc vector. AngII-induced increase in reporter luciferase activity was enhanced by *Snrk* silencing and attenuated by *Snrk* overexpression (Fig. 8e–h). Like AngII administration, overexpression of constitutively active p65 (p65-CA) increased luciferase activity from pGL3-TNF-α and pGL3-MCP-1 in GnECs transfected with control siRNA. Luciferase activity further increased in cells transfected with *Snrk* siRNA (Supplementary Fig. 16a and b).

Given that SNRK is a serine/threonine kinase, we also tested whether SNRK regulates p65 via its kinase activity, with SNRK activation via phosphorylation of the T-loop residue threonine-173^33^. AngII-induced phosphorylation of p65 in the presence of either WT SNRK or a kinase-dead SNRK mutant (SNRK-T173A), in which threonine-173 of the catalytic domain was replaced by an alanine (Supplementary Fig. 17a). In addition, overexpression of either WT SNRK or SNRK-T173A suppressed the expression of proinflammatory cytokines (Supplementary Fig. 17a and b), indicating that suppression of p65 by SNRK is independent of SNRK kinase activity.

## Discussion

In this study, we have uncovered a signaling pathway associated with AngII-induced renal injury. We found that AngII increased circulating miR-103a-3p, which selectively downregulated Snrk in renal endothelial cell, resulting in over-activation of NF-κB/p65 and consequent renal injury. Increased levels of circulating miR-103a-3p were found in hypertensive patients with renal injury and AngII-infused mice. Consistently, we observed that overexpression of miR-103a-3p aggravated renal injury in AngII-infused mice. Conversely, inhibition of miR-103a-3p with either LNA-anti-miR-103a-3p or AAV-anti-miR-103a-3p attenuated AngII-induced renal injury. Moreover, we found that miR-103a-3p selectively suppressed the expression of Snrk in GnECs, and that deletion of endothelial *Snrk* exacerbated AngII-induced renal injury. Finally, we found that downregulation of Snrk in GnECs resulted in over-activation of the NF-κB/p65 pathway, leading to aberrant renal inflammation and fibrosis. Overall, this study revealed that AngII causes renal injury via a signaling axis comprising circulating miR-103a-3p, endothelial SNRK, and p65. Our results suggest that targeting this axis represents a promising therapeutic strategy for treating AngII-induced renal injury and HN (Supplementary Fig. 18).

A key finding of the present study was that circulating miR-103a-3p plays a critical role in the AngII-induced renal injury. The global profile of circulating miRNAs demonstrates significant alterations under hypertensive conditions, with an elevation of miRNAs including miR-144-5p and miR-1825^15,16^ and downregulation of miRNAs including miR-140-3p, miR-532-3p, and miR-190^15,16,25^. Here, we found that increased circulating miR-103a-3p levels were associated with renal injury in hypertensive patients and AngII-infused mice. Although circulating miRNAs demonstrating differential expression are considered disease biomarkers, their roles, and functions in pathogenesis often remain unclear. Surprisingly, administration of LNA-anti-miR-103a-3p protected against AngII-induced renal injury, as evidenced by reduced proteinuria, kidney fibrosis, and inflammation. These findings were consistent with a previous report of anti-miR-103-induced suppression of the inflammatory response in vascular endothelial cells^34^. Conversely, overexpression of miR-103a-3p using AAV-miR-103a-3p further increased miR-103a-3p levels after AngII infusion and aggravated AngII-induced renal injury, as evidenced by increases in proteinuria, kidney fibrosis, and the inflammatory response. However, AAV-miR-103a-3p did not change the blood pressure or renal function in the absence of AngII. Interestingly, miR-103a-3p levels did not change in GnECs co-cultured with AngII. However, we observed increased miR-103a-3p levels in GnECs co-cultured with serum from AngII-infused mice. In addition, miR-103a-3p deficiency eliminated the inflammatory response of GnECs to AngII-infused mouse serum. Our results suggested that AngII increases levels of circulating miR-103a-3p, resulting in renal injury via the accumulation of miR-103a-3p in GnECs.

Also, an interesting finding was that Monopril (ACEi) or Diovan (β-blocker)-treated patients who had improved renal function with decreased ACR had profoundly reduced levels of miR-103a-3p in either urine or serum. In contrast, miR-103a-3p levels were not altered and higher in patients without renal improvement despite there were significant reductions of blood pressures in Diovan and Monopril-treated patients. Taken together, our data indicate that circulating miR-103a-3p levels are tightly linked to renal injury, but not to blood pressure elevation. The results suggest that circulating miR-103a-3p levels may function as a molecular biomarker for renal injury and work as a potential target for improving renal function, but not blood pressure.

Another important aspect of the present study was the identification of SNRK as a target of miR-103a-3p. A single miRNA can target multiple genes. Previous reports have identified various mRNAs as targets of miR-103a, including those encoding phosphatase and tensin homolog (PTEN)^35^, Kruppel-like factor 4 (KLF4)^34^, and a disintegrin and metalloproteinase domain-containing protein 10 (ADAM-10)^36^. In lung cancer cells, hypoxia-induced increase in miR-103a levels leads to the activation of AKT and signal transducer and activator of transcription 3 (STAT3) via inhibition of PTEN. We observed that miR-103a-3p negatively regulates *SNRK* expression in vitro and in vivo, with increased circulating miR-103a-3p levels associated with the downregulation of *SNRK* in human and mouse kidneys. We also observed a negative correlation between miR-103a-3p and Snrk in cultured GnECs. Experiments in vivo also demonstrated that miR-103a-3p levels negatively correlate with renal injury and with AngII-modulated *Snrk* expression in kidney tissues. Infusion of mice with AngII increased circulating miR-103a-3p levels and downregulated *Snrk* in GnECs. However, treatment of cultured GnECs with AngII did not decrease *Snrk* expression in the cells, suggesting a role for circulating miR-103a-3p. Indeed, we found that serum from AngII-infused mice suppressed *Snrk* expression in cultured GnECs in a manner dependent on miR-103a-3p, indicating that GnEC Snrk levels are regulated by circulating miR-103a-3p.

Our findings demonstrated the pivotal role that Snrk plays in AngII-induced renal injury. SNRK is a member of the adenosine monophosphate (AMP)-activated protein kinase (AMPK) family^37–40^. SNRK is highly expressed in heart, brain, blood vessel, kidney, and adipose tissues. Global homozygous *Snrk* knockout mice die at birth^39^, and myocardial *Snrk* deletion leads to cardiac failure and death by 8–10 months of age, possibly resulting from cardiac mitochondrial dysfunction^38^. Although in adipocytes, *Snrk* deficiency has been shown to induce an inflammatory response mediated by an increase in Tnf-α and Il-6^40^, to date, the role of Snrk in the development of renal injury has remained unclear. Presently, we observed an inverse correlation between Snrk levels and the severity of renal damage caused by AngII. Similarly, AngII infusion increased blood pressure, reduced renal *Snrk* expression, and induced renal injury in mice. The finding of endothelial *Snrk* deletion exacerbating AngII-induced renal injury substantially underscored the protective role of Snrk against the development of AngII-induced renal injury. The protective role  of Snrk is likely dependent on its interaction with NF-κB/p65, a critical transcriptional factor mediating the genesis of renal damage caused by AngII infusion^32^. Under physiological conditions, p65 mainly localizes in the cytoplasm. Upon phosphorylation, it is activated and translocated to the nucleus, forming a complex with Snrk. Snrk levels inversely correlated with the AngII-induced activity of proinflammatory gene promoters, suggesting that Snrk may regulate p65 transcriptional activity. Consistent with our findings, endothelium NF-κB/p65 activation has been implicated in AngII-induced renal damage^32^. Thus, Snrk likely inhibits AngII-induced renal injury by modulating the NF-κB/p65 pathway.

In summary, our findings reveal a previously unidentified mechanism by which AngII induces renal injury, with circulating miR-103a-3p-reduced glomerular endothelial Snrk expression. In turn, Snrk can bind p-p65 to regulate its transcriptional activity and control the AngII-induced renal damage. We demonstrate that renal injury can be regulated by modulating miR-103a-3p and Snrk levels. Thus, our data suggest that targeting the circulating miR-103a-3p/endothelial Snrk/p65 signaling axis represents a potential treatment strategy for AngII-induced renal injury.

## Methods

### Animals

Endothelial cell-specific deletion of *Snrk* (*Snrk*^f/f^/Cre^VE-Cadh+/−^) mice were generated by crossbreeding *Snrk*-floxed (*Snrk*^f/f^) mice^39^ (kindly provided by Dr. Ramani Ramchandran at Medical College of Wisconsin) with vascular endothelial cadherin (VE-cadh)-Cre transgenic mice with a C57BL/6J background (Jackson Laboratories, Bar Harbor, ME). The animals were housed in a controlled environment (20 ± 2 °C, 12-h/12-h light/dark cycle) and were free access to water and a standard chow diet. All animal experimental protocols were approved by the Institutional Animal Care and Use Committee at Georgia State University and conformed to NIH guidelines. We have complied with all relevant ethical regulations.

### AngII-induced chronic kidney disease

*Snrk*^f/f^/Cre^VE-Cadh+/−^, *Snrk*^wt/wt^/Cre^VE-Cadh+/−^, and C57BL/6J mice were used for the experiments. Alzet osmotic mini-pumps (Durect Corporation, CA) loaded with vehicle or AngII (Sigma-Aldrich, MO) were implanted subcutaneously in the dorsal region under isoflurane anesthesia to maintain a delivery rate of 1 mg/kg per day for a course of 4 weeks. Blood pressure was measured by carotid artery cannulation as previously described^41^. A PE-10 catheter was inserted into the right carotid artery, and arterial blood pressure was monitored with a pressure transducer and recorded by PowerLab. At the end of the experiments mice were weighted and euthanized, and kidneys were immediately excised, weighed, and processed for tissue analysis.

For LNA-anti-miRNA experiments, 1 day after mini-pump implantation, mice (*n* = 8 mice per group) received two intravenous injections of 25 mg kg^−1^ of LNA inhibitors (LNA-anti-miR-Control or LNA-anti-miR-103a-3p) per week for 3 weeks^42–44^. The samples were collected 4 weeks after mini-pump implantation. LNA-anti-miR-103-3p (Cat, YI04107447) and LNA-anti-Control (Cat, YI00199006) were from Qiagen.

### Human samples

We collected urine and serum samples from 60 newly diagnosed hypertensive patients (SBP > 130 mm Hg) and 18 individuals with normal blood pressure (SBP < 130 mm Hg). Thirty-one patients with hypertensive nephropathy (HN, *n* = 31) out of 60 patients received no medical treatment while 29 patients with renal dysfunctions were treated with either Diovan (β-blockers) (*n* = 13) or Monopril (ACEi) (*n* = 16) for 2–3 weeks. The recruitment criteria for HN were as follows: (1) evidence of hypertension (blood pressure above 130/90 mm Hg) before the detection of proteinuria, hematuria and/or impaired renal function; (2) evidence from renal pathology in accordance with WHO criteria^45^ (The main diagnostic features were intimal thickening, medial hypertrophy, reduplication of the internal elastic lamina and glomerular sclerosis); (3) participates were between 18 and 60 years of age; (4) individuals were excluded from the study if they had other glomerular diseases or systemic disorders, such as glomerulonephritis or diabetic nephropathy, and gout or urinary stones^46,47^. The healthy control samples were from individuals with normal blood pressure and renal function. The human subject studies were approved and performed according to the standards established by the Ethics Committee on Human Subject Research at Tianjin Medical University. We have complied with all relevant ethical regulations. Written informed consent was received from all human participants prior to inclusion in the study.

### Isolation and culture of glomerular endothelial cells (GnECs)

Mouse GnECs were isolated and cultured as previously described^9^. Briefly, kidney from 2- to 3-day‐old mice was cut into small fragments and digested with 2.5 mL 0.1% collagenase type II (Worthington Biochemical Corp., NJ) at 37 °C for 45 min. After digestion, ice-cold PBS was added to the cell suspension and the suspension was filtered sequentially through a 70-μm and a 40-μm cell strainer. The cell suspension was incubated with Dynabeads (#110.07, Thermo Fisher Scientific, MA) precoated with platelet endothelial cell adhesion molecule-1 (PECAM-1) antibody (#553369, BD Pharmingen, CA) at room temperature for 10 min, and then the magnetic beads were collected with a magnetic separator. The magnetic beads were resuspended and cultured for 7–10 days in culture flasks using EBM medium with Endothelial Cell Growth Medium (EGM) Single Quotes. The cells were maintained in EBM medium supplemented with penicillin and streptomycin (1%) and fetal bovine serum (FBS) (10%). Until approaching confluence around 90%, the cells were detached with Trypsin: EDTA (0.05%: 0.5 M) and resorted by Dynabeads (Dynal #110.07) precoated with intercellular adhesion molecule 2 (ICAM-2) antibody (#553325, BD Pharmingen, CA).

### Cell culture and transfection

MiRNA negative control (miR-con and anti-miR-con), miR-103a-3p mimics, and anti-miR-103a-3p were obtained from Thermo Fisher Scientific (miR-103a-3p mimics, Cat. # 4464066; anti-miR-103a-3p, Cat. # AM17000; miR-con, Cat. # 4464058; anti-miR-con, Cat. # AM17010, MA). Isolated mouse GnECs were cultured in the EGM-2 medium containing 5% (v/v) fetal bovine serum (FBS) and EGM-2 Single Quots (Lonza, GA). GnECs were transfected with miRNA or plasmids using the electroporation P5 Primary Cell Nucleofector Kits (Lonza, GA). The transfection efficiency of miRNA or plasmids was determine by measuring target gene expression by real-time polymerase chain reaction (PCR).

### Urine and blood analysis

At the end of the experiments, urine was collected for 24 h using a metabolic cage, the concentrations of urinary albumin were measured by Elisa (Nephrat, Exocell, Philadelphia, PA, USA). Spot urine samples were also collected to assess the urinary albumin-to-creatinine ratio (ACR) using a Mouse Albumin ELISA Quantification Set (Cat. # E90-134, Bethyl Laboratories, Montgomery, TX). Creatinine concentrations in plasma and urine were measured using the detection kits (Arbor Assays, Cat. # KB02-H1) according to the manufacturer’s instructions.

### Adeno-associated virus (AAVs) preparation

The AAV system was modified and adjusted to express miR-103a-3p in vivo. According to the mature sequence of miR-103a-3p in miRBase, the oligonucleotides for the expression of miR-103a-3p, anti-miR-103a-3p, and miR-random were listed in Supplementary Table 2. The AAV9 was packaged by triple plasmids cotransfection in HEK293 cells (ATCC) and purified as described previously^48–50^. For in vivo experiments, 1 day after mini-pump implantation, mice were injected with AAVs (5 × 10^11^ genome copies per mouse) via tail vein, respectively, as described previously^51–53^.

### miRNA FISH

Mouse and human tissues were collected and fixed with 3.7% paraformaldehyde (v/v)^43,54,55^. Briefly, deparaffinized sections were treated with proteinase-K (25 µg mL^−1^ for 8 min at 37 °C). Double-FAM-labeled miR-103a-3p probe (mmu-miR-103a-3p miRCURY LNA miRNA Detection probe, Cat YD00615519, Exiqon, Denmark) was incubated at 30 nM for 1 h in Exiqon hybridization buffer (Exiqon) at 57 °C. Polyclonal sheep anti-vWF (ab11713, 1:100, Abcam, Cambridge, UK) was incubated at room temperature and detected with Alexa Fluor 555-conjugated donkey anti-Sheep (1:400, Thermo Fisher Scientific, Waltham, MA). And polyclonal Rabbit anti-SNRK (1:100, ab96762, Abcam, Cambridge, UK) was incubated at room temperature and detected with Alexa Fluor 488-conjugated goat anti-Rabbit (1: 400, Thermo Fisher Scientific, Waltham, MA). Slides were mounted with Anti-fade Gold with DAPI (Invitrogen). Image acquisition was performed with confocal microscope (LSM800, Carl Zeiss Microscopy Ltd, Cambridge, MA). The colocalization coefficient was quantified using Zen 2011 software. For each channel, the colocalization coefficient was calculated as the ratio of pixels exhibiting colocalized SNRK to total pixels exhibiting vWF staining. All quantitation was done using original, unmodified data images.

### Plasmid construction

The cDNA for human *SNRK* was obtained using RT-PCR with total RNA samples from human umbilical vein endothelial cells (HUVECs) (Lonza, GA). The PCR product was digested with EcoR I and Xho I, endonuclease restriction sites were designed into the PCR primers, and the product amplified using these primers were cloned into the vector pCMV-HA, resulting in a mammalian expression construct for SNRK (pCMV-SNRK-HA). The entire insert in the SNRK expression construct was sequenced and verified. The mutant for the kinase-inactive SNRK-T173A (pCMV-SNRK-T173A-HA) was generated using Q5 Site-Directed Mutagenesis Kit (Promega, WI). The primers were listed in Supplementary Table 2.

### miRNA isolation and detection

Total urinary microRNAs were purified from collected urine using Urine microRNA Purification Kit (Cat. #29000, Norgen Biotek Corp, Ontario, Canada) according to the manufacturer’s instruction. Total circulating microRNAs were isolated using miRNeasy Serum/Plasma Kit (QIAGEN, German) following the manufacturer’s instruction. Total microRNAs from kidney tissues and cells were purified using mirVana miRNA Isolation Kit (Thermo Fisher Scientific, Waltham, MA). A synthetic miRNA [*Caenorhabditis elegans* cel-miR-39 (5′-UCACCGGGUGUAAAUCAGCUUG-3′)] was added to the serum aliquots as an exogenous miRNA spiked-in control to allow for normalization of sample-to-sample variation in the RNA isolation and synthetic procedure. Moreover, *U6* snRNA was used as endogenous miRNA control. Real-time PCR was performed using CEF96 Real-Time PCR Detection System (Bio-Rad, CA). The primers for miR-103a-3p and U6 were purchased from ABM (Vancouver, Canada) (hsa-miR-103a-3p, Cat. # MPH02063; mmu-miR-103a-3p, Cat. # MPM00763; human U6, Cat. # MPM00001; mouse U6, Cat. # MPM00002; cel-miR-39, Cat. # MPH00006). MiR-103a-3p expression was normalized using the 2^−ΔΔΔCt^ method from the Ct values of the miR-103a-3p relative to the combination of endogenous and an exogenous control miRNA (ΔCt = Ct_cel-miR-39_ – Ct_*U6* snRNA_)^56,57^.

### Quantitative real-time PCR

Total mRNAs from tissues or cells were extracted using TRIzol reagent (Invitrogen), then reverse-transcribed into cDNA using the iScript cDNA Synthesis Kit (Bio-Rad, Hercules, CA), following the manufacturer’s protocol. Q-PCR was performed using CEF96 Real-Time PCR Detection System (Bio-Rad, CA). The primers used for mRNA quantification were listed in Supplementary Table 3. Housekeep gene *GAPDH* was as the internal control. The relative expression levels of the genes were calculated using the 2^−∆∆CT^ method.

### Histological analysis

Renal fibrosis was assessed by staining renal sections with Masson’s trichrome. The glomerular collagen content was quantified by measuring the ratio of blue staining area to total glomerular area.

### Immunohistochemistry

Kidney sections (4 µm) were deparaffinized and rehydrated. Endogenous peroxidase activity was blocked using 0.3% H_2_O_2_ at 37 °C for 10 min. Heat-mediated antigen retrieval was performed using 10% citrate buffer. Then the sections were incubated with primary antibodies followed by secondary antibody and visualized with diaminobenzydine (DAB). Primary antibodies and dilutions used were as follows: Col IV (1:200; ab6586, Abcam), SNRK (1:500; ab96762, Abcam). The stained slides were photographed using an OLYMPUS BX51 microscope and the percentages of positive cells and staining intensities were scored as previously described^58^.

### Western blotting analysis

Western blotting was performed as previously described^59^. Proteins were extracted from cells/tissue using RIPA lysis buffer. The protein concentrations were assayed by bicinchoninic acid method. Equal amounts of protein (20–40 μg) was separated by SDS-PAGE and then transferred electrophoretically to polyvinylidene fluoride membranes. Membrane was incubated with a 1:1000 dilution of primary antibody at 4 °C overnight, followed by a 1:5000 dilution of horseradish peroxidase-conjugated secondary antibody. Protein bands were visualized by enhanced chemoluminescence system. Primary antibodies and dilutions used were as follows: SNRK (1:1000; ab96762, Abcam), MCP-1 (1:1000; ab7202, Abcam), TNF-α (1;1000; 3703, Cell Signal Technology), Col I (1:1000; 84336, Cell Signal Technology), and Col IV (1:200; ab6586, Abcam). GAPDH was used as an internal reference. The image collection and quantitative analysis were performed using Quantity One software (Bio-Rad Laboratories, Hercules, CA USA). Uncropped immunoblot gels are displayed in Supplementary Fig. 19.

### Immunofluorescence

Slides were washed with PBS and fixed with acetone for 10 min and then blocked with goat serum, and incubated with primary antibodies (anti-vWF, ab11713 from Abcam, 1:100; anti-SNRK from Abcam, 1:100) overnight at 4 °C. After three PBS washes, the slides were incubated with secondary antibody Alexa 555 goat anti-rabbit and Alexa 488 goat anti-rat for 1 h at 37 °C. DAPI was used to counterstain the nuclei, and then washed and observed under a fluorescence microscope IX71 (Olympus, Japan).

### Luciferase reporter assays

To elucidate the putative complementary sequences of miR-103a-3p identified in the 3′UTR of *SNRK* mRNA, we cloned the two different parts (50−387 bp and 1457−2157 bp) of 3′UTR of SNRK mRNA into the vector pMIR, constructing two different luciferase plasmids pMIR-167 and pMIR-1994, respectively. The PCR product was digested with Hind III and Sac I and endonuclease restriction sites were designed into the PCR primers. The putative binding sites of miR-103a-3p, which were located at 167 and 1994 bp of the human *SNRK* mRNA, were deleted by Q5 Site-Directed Mutagenesis Kit (Promega, WI), resulting in two mutated *SNRK*, pMIR-167-Δ and pMIR-1994-Δ. These plasmids were sequenced and verified. HEK293 cells were cotransfected with miRNA, luciferase plasmids, and internal control Renilla luciferase plasmid. The relative luciferase activities were evaluated with a Dual Luciferase Reporter Assay Kit (Promega, WI). The pGL3 luciferase Reporter Vectors (Promega, WI) were used for constructing luciferase vectors. As previously reported, the PCR product of the promoter of MCP-1 (from −3555 bp to +74 bp) was cloned into pGL vector to construct pGL-MCP-1. The sequence of human TNF-α promoter (−1173 bp to +130 bp) was cloned and then inserted into pGL vector to generate pGL-TNF-α. All the primers for plasmids were listed in Supplementary Table 2. The plasmids were verified by DNA sequencing. GnECs were transfected with these plasmids using the electroporation P5 Primary Cell Nucleofector Kits (Lonza, GA) according to the manufacturer’s protocol. The relative luciferase activities were evaluated with a Dual Luciferase Reporter Assay Kit (Promega, WI).

### Statistical analysis

All the data were expressed as means ± standard error of the mean (SEM), and differences between two groups were analyzed by Student’s *t*-test, whereas comparisons among more than two groups were evaluated with a one-way analysis of variance followed by a Bonferroni post hoc test or by a two-way ANOVA analysis using SPSS version 20. *P* values <0.05 was considered statistically significant.

### Reporting summary

Further information on research design is available in the Nature Research Reporting Summary linked to this article.

## Supplementary information

Supplementary Information

Reporting Summary

Source Data

## Data Availability

All data are available from the corresponding author upon reasonable request. The source data underlying all main and supplementary figures are available as a Source Data file.

## References

[1] Francois H (2004). Prevention of renal vascular and glomerular fibrosis by epidermal growth factor receptor inhibition. FASEB J..

[2] Mennuni S (2014). Hypertension and kidneys: unraveling complex molecular mechanisms underlying hypertensive renal damage. J. Hum. Hypertens..

[3] Iversen BM, Ofstad J (1987). The effect of hypertension on glomerular structures and capillary permeability in passive Heymann glomerulonephritis. Microvasc. Res..

[4] Wang PR, Kitamura H, Shimizu A, Yamanaka N (2015). Glomerular damage in experimental proliferative glomerulonephritis under glomerular capillary hypertension. Kidney Blood Press. Res..

[5] Mezzano SA, Ruiz-Ortega M, Egido J (2001). Angiotensin II and renal fibrosis. Hypertension.

[6] Murphy AM, Wong AL, Bezuhly M (2015). Modulation of angiotensin II signaling in the prevention of fibrosis. Fibrogenesis Tissue Repair.

[7] Kurts C, Panzer U, Anders HJ, Rees AJ (2013). The immune system and kidney disease: basic concepts and clinical implications. Nat. Rev. Immunol..

[8] Kitching AR, Hutton HL (2016). The players: cells involved in glomerular disease. Clin. J. Am. Soc. Nephrol..

[9] Imig JD, Ryan MJ (2013). Immune and inflammatory role in renal disease. Compr. Physiol..

[10] Wang XC (2013). Clinical and pathological analysis of the kidney in patients with hypertensive nephropathy. Exp. Ther. Med..

[11] Brewster UC, Perazella MA (2004). The renin-angiotensin-aldosterone system and the kidney: effects on kidney disease. Am. J. Med.

[12] Bartel DP (2009). MicroRNAs: target recognition and regulatory functions. Cell.

[13] Kriegel AJ (2015). Endogenous microRNAs in human microvascular endothelial cells regulate mRNAs encoded by hypertension-related genes. Hypertension.

[14] Baker MA (2017). Tissue-specific microRNA expression patterns in four types of kidney disease. J. Am. Soc. Nephrol..

[15] Muralidharan J (2017). Extracellular microRNA signature in chronic kidney disease. Am. J. Physiol. Ren. Physiol..

[16] Rudnicki M (2016). Renal microRNA- and RNA-profiles in progressive chronic kidney disease. Eur. J. Clin. Investig..

[17] Huttenhofer A, Mayer G (2017). Circulating miRNAs as biomarkers of kidney disease. Clin. Kidney J.

[18] Rysz J, Gluba-Brzozka A, Franczyk B, Jablonowski Z, Cialkowska-Rysz A (2017). Novel biomarkers in the diagnosis of chronic kidney disease and the prediction of its outcome. Int. J. Mol. Sci..

[19] Zernecke A (2009). Delivery of microRNA-126 by apoptotic bodies induces CXCL12-dependent vascular protection. Sci. Signal.

[20] Vickers KC, Palmisano BT, Shoucri BM, Shamburek RD, Remaley AT (2011). MicroRNAs are transported in plasma and delivered to recipient cells by high-density lipoproteins. Nat. Cell Biol..

[21] Tabet F (2014). HDL-transferred microRNA-223 regulates ICAM-1 expression in endothelial cells. Nat. Commun..

[22] Deng X (2017). Circulating miRNA-24 and its target YKL-40 as potential biomarkers in patients with coronary heart disease and type 2 diabetes mellitus. Oncotarget.

[23] Sidaway P (2016). Prostate cancer: circulating miRNAs indicate high-risk disease. Nat. Rev. Urol..

[24] de Souza PS, Faccion RS, Bernardo PS, Maia RC (2016). Membrane microparticles: shedding new light into cancer cell communication. J. Cancer Res. Clin. Oncol..

[25] Mao L (2016). Exosomes decrease sensitivity of breast cancer cells to adriamycin by delivering microRNAs. Tumour Biol.: J. Int. Soc. Oncodev. Biol. Med..

[26] Karolina DS (2012). Circulating miRNA profiles in patients with metabolic syndrome. J. Clin. Endocrinol. Metab..

[27] Bacon S (2015). MicroRNA-224 is readily detectable in urine of individuals with diabetes mellitus and is a potential indicator of beta-cell demise. Genes.

[28] Zhang Z, Wu S, Muhammad S, Ren Q, Sun C (2018). miR-103/107 promote ER stress-mediated apoptosis via targeting the Wnt3a/beta-catenin/ATF6 pathway in preadipocytes. J. Lipid Res..

[29] Xu MC (2015). miR-103 regulates oxidative stress by targeting the BCL2/adenovirus E1B 19 kDa interacting protein 3 in HUVECs. Oxid. Med. Cell. Longev..

[30] Agarwal, V., Bell, G. W., Nam, J. W. & Bartel, D. P. Predicting effective microRNA target sites in mammalian mRNAs. *eLife* **4**, e05005 (2015). 10.7554/eLife.05005 PMC4532895 26267216

[31] Leelahavanichkul A (2010). Angiotensin II overcomes strain-dependent resistance of rapid CKD progression in a new remnant kidney mouse model. Kidney Int.

[32] Henke N (2007). Vascular endothelial cell-specific NF-κB suppression attenuates hypertension-induced renal damage. Circ. Res.

[33] Jaleel M (2005). Identification of the sucrose non-fermenting related kinase SNRK, as a novel LKB1 substrate. FEBS Lett..

[34] Hartmann P (2016). Endothelial Dicer promotes atherosclerosis and vascular inflammation by miRNA-103-mediated suppression of KLF4. Nat. Commun..

[35] Hsu YL (2018). Hypoxic lung-cancer-derived extracellular vesicle microRNA-103a increases the oncogenic effects of macrophages by targeting PTEN. Mol. Ther..

[36] Jiao T (2017). Role of microRNA-103a targeting ADAM10 in abdominal aortic aneurysm. Biomed. Res. Int..

[37] Kertesz N, Samson J, Debacker C, Wu H, Labastie MC (2002). Cloning and characterization of human and mouse SNRK sucrose non-fermenting protein (SNF-1)-related kinases. Gene.

[38] Rines AK (2017). Snf1-related kinase improves cardiac mitochondrial efficiency and decreases mitochondrial uncoupling. Nat. Commun..

[39] Cossette SM (2014). Sucrose non-fermenting related kinase enzyme is essential for cardiac metabolism. Biol. Open.

[40] Li Y (2013). Identification of sucrose non-fermenting-related kinase (SNRK) as a suppressor of adipocyte inflammation. Diabetes.

[41] Zhang W (2014). Endothelial cell-specific liver kinase B1 deletion causes endothelial dysfunction and hypertension in mice in vivo. Circulation.

[42] Zhang WC (2016). Tumour-initiating cell-specific miR-1246 and miR-1290 expression converge to promote non-small cell lung cancer progression. Nat. Commun..

[43] Elmen J (2008). Antagonism of microRNA-122 in mice by systemically administered LNA-antimiR leads to up-regulation of a large set of predicted target mRNAs in the liver. Nucleic acids Res..

[44] Caballero-Garrido E (2015). In vivo inhibition of miR-155 promotes recovery after experimental mouse stroke. J. Neurosci..

[45] Luke RG (1999). Hypertensive nephrosclerosis: pathogenesis and prevalence. Essential hypertension is an important cause of end-stage renal disease. Nephrol. Dial. Transplant..

[46] Innes A, Johnston PA, Morgan AG, Davison AM, Burden RP (1993). Clinical features of benign hypertensive nephrosclerosis at time of renal biopsy. Q. J. Med..

[47] Zucchelli P, Zuccala A (1993). The diagnostic dilemma of hypertensive nephrosclerosis: the nephrologist’s view. Am. J. Kidney Dis..

[48] Inagaki K (2006). Robust systemic transduction with AAV9 vectors in mice: efficient global cardiac gene transfer superior to that of AAV8. Mol. Ther..

[49] Bostick B, Ghosh A, Yue Y, Long C, Duan D (2007). Systemic AAV-9 transduction in mice is influenced by animal age but not by the route of administration. Gene Ther..

[50] Nakai H (2005). Unrestricted hepatocyte transduction with adeno-associated virus serotype 8 vectors in mice. J. Virol..

[51] Song E (2003). RNA interference targeting Fas protects mice from fulminant hepatitis. Nat. Med.

[52] Li H (2016). MicroRNA-21 lowers blood pressure in spontaneous hypertensive rats by upregulating mitochondrial translation. Circulation.

[53] Zhang F (2005). Long-term modifications of blood pressure in normotensive and spontaneously hypertensive rats by gene delivery of rAAV-mediated cytochrome P450 arachidonic acid hydroxylase. Cell Res..

[54] Nielsen BS (2014). miR-21 expression in cancer cells may not predict resistance to adjuvant trastuzumab in primary breast cancer. Front. Oncol..

[55] Maegdefessel L (2012). Inhibition of microRNA-29b reduces murine abdominal aortic aneurysm development. J. Clin. Invest.

[56] Schwarzenbach H, da Silva AM, Calin G, Pantel K (2015). Data Normalization Strategies for MicroRNA Quantification. Clin. Chem..

[57] Sourvinou IS, Markou A, Lianidou ES (2013). Quantification of circulating miRNAs in plasma: effect of preanalytical and analytical parameters on their isolation and stability. J. Mol. Diagn..

[58] Ding Y (2016). AMP-Activated Protein Kinase Alpha 2 Deletion Induces VSMC Phenotypic Switching and Reduces Features of Atherosclerotic Plaque Stability. Circ. Res.

[59] Ding Y, Chen J, Okon IS, Zou MH, Song P (2016). Absence of AMPKalpha2 accelerates cellular senescence via p16 induction in mouse embryonic fibroblasts. Int. J. Biochem. cell Biol..

