# Publisher Correction: Circulating miR-103a-3p contributes to angiotensin II-induced renal inflammation and fibrosis via a SNRK/NF-κB/p65 regulatory axis

**DOI:** 10.1038/s41467-019-11515-z

**Published:** 2019-08-06

**Authors:** Qiulun Lu, Zejun Ma, Ye Ding, Tatiana Bedarida, Liming Chen, Zhonglin Xie, Ping Song, Ming-Hui Zou

**Affiliations:** 1 Center for Molecular and Translational Medicine, Georgia State University, Atlanta, GA USA; 2 Key Laboratory of Hormones and Development (Ministry of Health), Tianjin Key Laboratory of Metabolic Diseases, Tianjin Metabolic Diseases Hospital & Tianjin Institute of Endocrinology, Tianjin Medical University, Tianjin, China

**Keywords:** miRNAs, Renal fibrosis

Correction to: *Nature Communications* 10.1038/s41467-019-10116-0, published online 13 May 2019.

The original version of this Article contained an error in Fig. 1 in which Fig. 1b was inadvertently replaced by a duplicate of Fig. 1a. This has been corrected in the PDF and HTML versions of the Article.

