# Supplementary material for: Circulating miR-103a-3p contributes to angiotensin II-induced renal inflammation and fibrosis via a SNRK/NF-κB/p65 regulatory axis
Source: Nat Commun. 2019 May 13;10:2145. doi: 10.1038/s41467-019-10116-0 (PMC6513984; doi:10.1038/s41467-019-10116-0)
Supplement: Supplementary file 1 — Supplementary Information [file 41467_2019_10116_MOESM1_ESM.docx]

**SUPPLEMENTARY INFORMATION**

**Circulating miR-103a-3p contributes to angiotensin II-induced renal inflammation and fibrosis via a SNRK/NF-κB/p65 regulatory axis**

**Lu** *et al*.

**
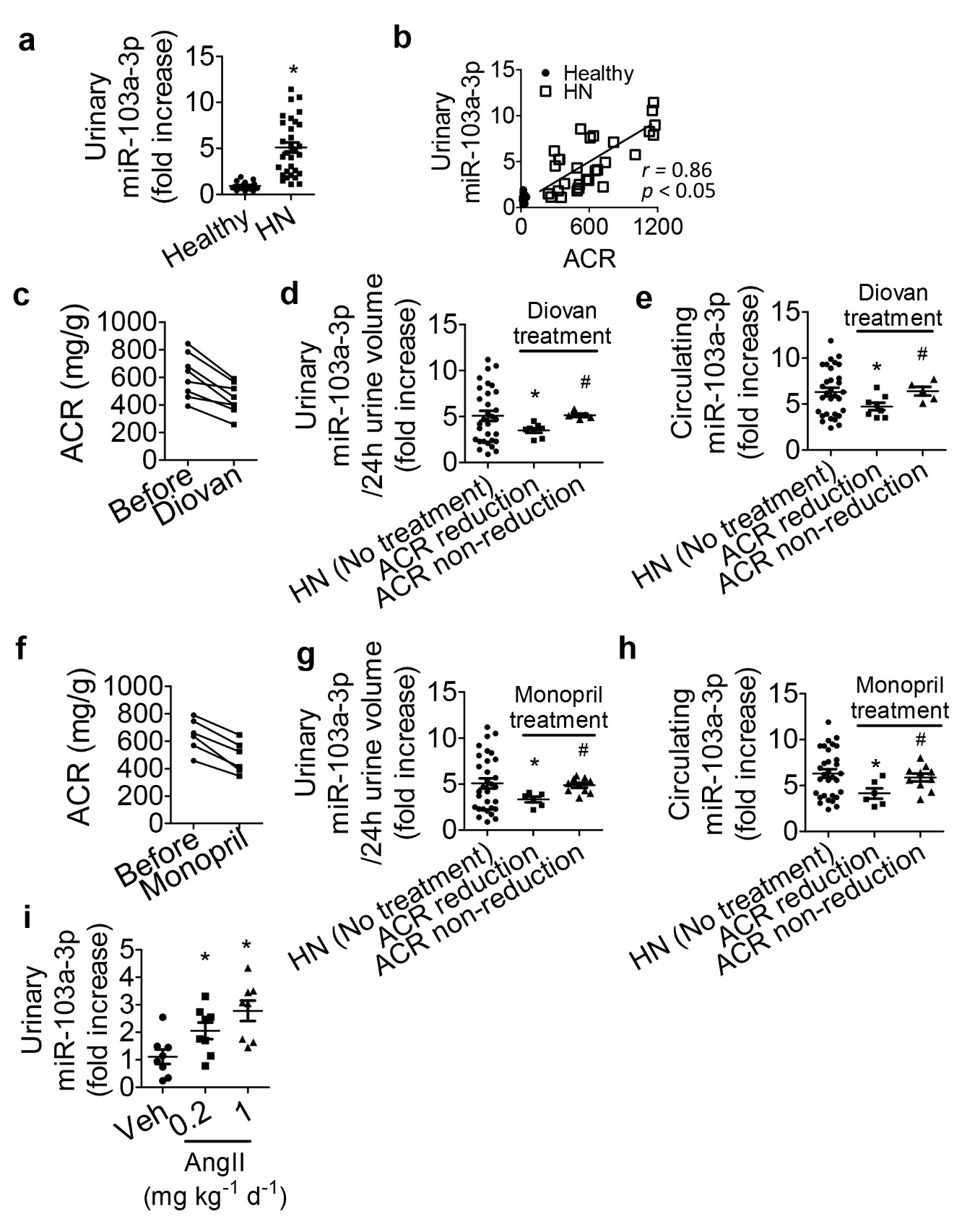
Supplementary Figure 1. Up-regulation of urinary miR-103a-3p *in vivo*. a** Urinary miR-103a-3p levels in healthy controls and HN patients. **b** Correlation between ACR and urinary miR-103a-3p levels in healthy individuals and HN patients. **p* < 0.05 relative to healthy controls. **c**-**h** Urine and serum samples from 60 newly diagnosed hypertensive patients (SBP>130) and 18 normal blood pressure individual (SBP<130) were collected. 31 out of 60 patients received no medical treatment while 29 patients with renal dysfunctions were treated with either Diovan (β-blockers) (n= 13) or Monopril (ACEi) (n = 16) for 2-3 weeks. 8 out of 13 Diovan-treated patients who had improved renal function with decreased ACR (**c**) had significantly reduced levels of miR-103a-3p levels in either urine (**d**) or serum (**e**); consistently, 6 out of 16 Monopril (ACEi)-treated patients who had improved renal function with decreased ACR (**f**) had reduced levels of miR-103a-3p in either urine (**g**) or serum (**h**). **p* < 0.05 relative to HN, #*p* < 0.05 relative to ACR reduction (Student’s *t*-test). **i** WT mice were infused with vehicle or AngII at two different doses (0.2 and 1 mg kg^-1^ d^-1^) for 4 weeks (n = 8, each group). Urinary miR-103a-3p levels were measured in these group mice. **p* < 0.05, relative to vehicle control. Each symbol represents an individual sample. Statistical analysis was carried out with one-way analysis of variance. The corresponding source data are available in the Source Data file.

**
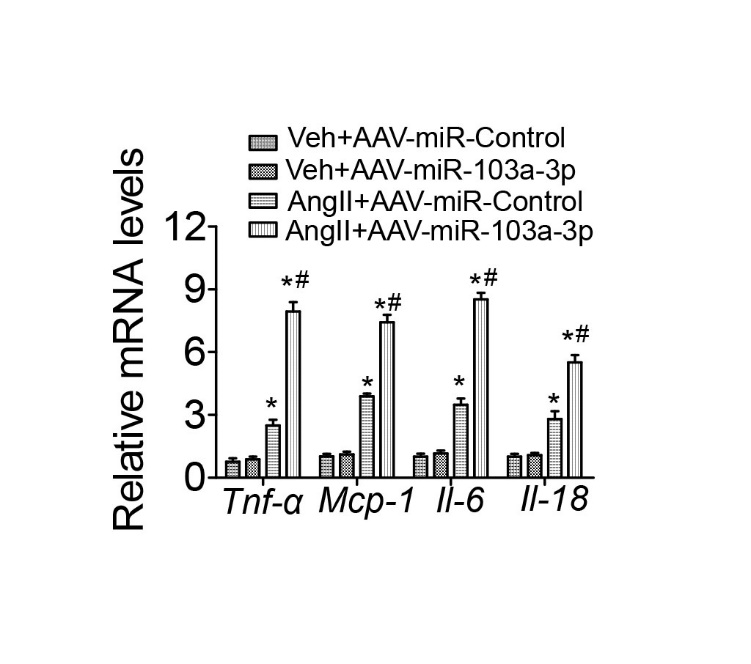
**

**Supplementary Figure 2. Up-regulation of proinflammatory factors after miR-103a-3p overexpression *in vivo*.** Mice transfected with AAV-miR-103a-3p or AAV-miR-control were infused with Veh or AngII (1 mg kg^-1^ d^-1^) for 4 weeks. The mRNA levels of *Mcp-1*, *Tnf-α*, *Il-6*, *Il-18* in kidneys from these four group mice were showed. For all experiments, n = 6 per group; **p* < 0.05 relative to vehicle control; ^#^*p* < 0.05 relative to AAV-miR-Control/AngII. Statistical analysis was carried out with a Student’s two-tailed *t*-test. The corresponding source data are available in the Source Data file.

**
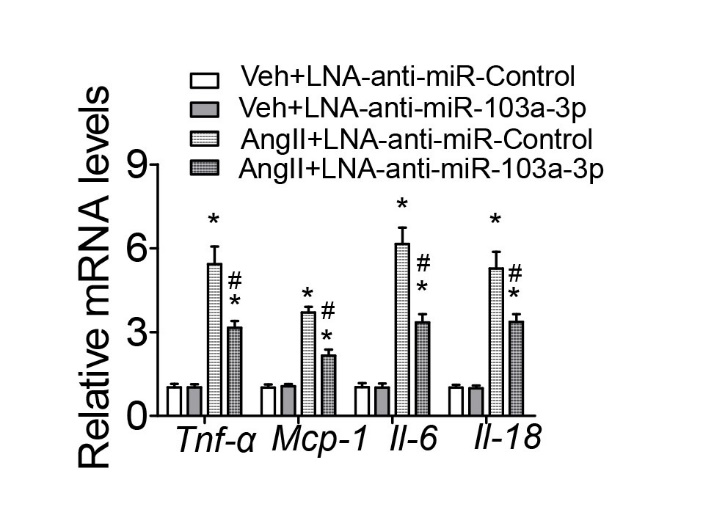
**

**Supplementary Figure 3. Suppression of proinflammatory factors after miR-103a-3p inhibition.** Mice injected with LNA-anti-miR-103a-3p or LNA-anti-miR-control were infused with Veh or AngII (1 mg kg^-1^ d^-1^) for 4 weeks. The mRNA levels of *Mcp-1*, *Tnf-α*, *Il-6*, *Il-18* in kidneys from these four group mice were showed. For all experiments, n = 8 per group; **p* < 0.05 relative to vehicle control; ^#^*p* < 0.05 relative to LNA-anti-miR-Control/AngII. Statistical analysis was carried out by a Student’s two-tailed *t*-test. The corresponding source data are available in the Source Data file.

**
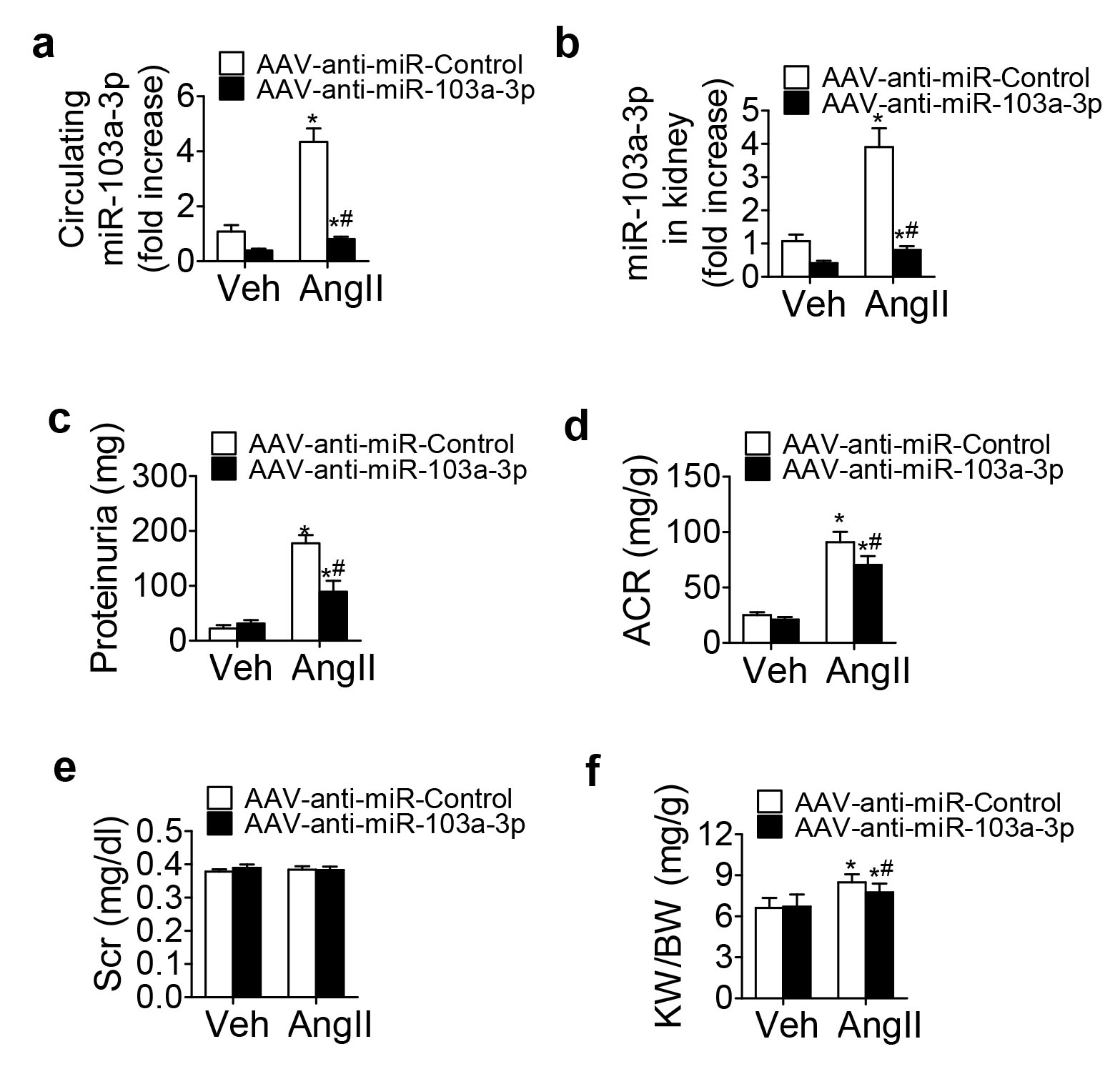
**

**Supplementary Figure 4. Reduction of miR-103a-3p ameliorates AngII‐induced renal dysfunction.** Mice transfected with AAV-anti-miR-103a-3p or AAV-anti-miR-control were infused with Veh or AngII (1 mg kg^-1^ d^-1^) for 4 weeks. **a** Comparison of serum miR-103a-3p levels. Serum miR-103a-3p levels were analyzed by qRT-PCR. **b** Comparison of miR-103a-3p levels in kidney tissues. **c** Albumin excretion rate comparison. **d** ACR comparison. **e** Serum creatinine comparison. **f** KW/BW comparison. For all experiments, n = 6 per group; **p* < 0.05 relative to vehicle control; ^#^*p* < 0.05 relative to AAV-anti-miR-Control. Statistical analysis was carried out by a Student’s two-tailed *t*-test. The corresponding source data are available in the Source Data file.

**
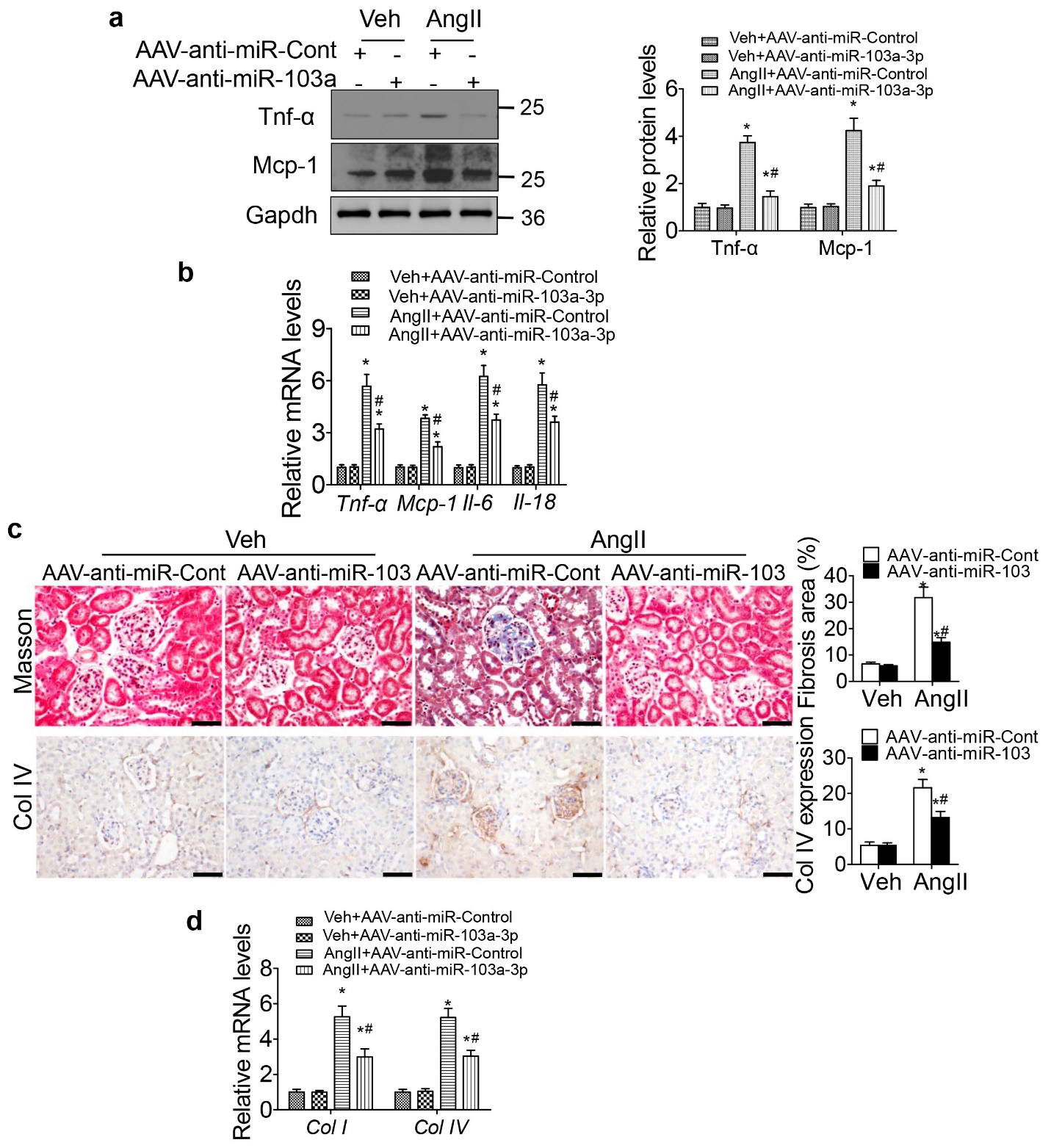
**

**Supplementary Figure 5. miR-103a-3p reduction ameliorates AngII‐induced renal inflammation and fibrosis.** Mice transfected with AAV-anti-miR-103a-3p or AAV-anti-miR-control were infused with Veh or AngII for 4 weeks. **a** Western blot analysis of Mcp-1 and Tnf-α expression in kidneys. **b** The mRNA levels of *Mcp-1*, *Tnf-α*, *Il-6*, *Il-18* in kidneys. **c** Representative images of trichrome staining and immunohistochemical staining of Col IV in kidneys (scale bar=50 µm). **d** *Col I* and *Col IV* mRNA levels were measured using qRT-PCR. For all experiments, n = 6 per group; **p* < 0.05 relative to vehicle control; ^#^*p* < 0.05 relative to AngII+AAV-anti-miR-Control. Statistical analysis was carried out by a Student’s two-tailed *t*-test. The corresponding source data are available in the Source Data file.

**
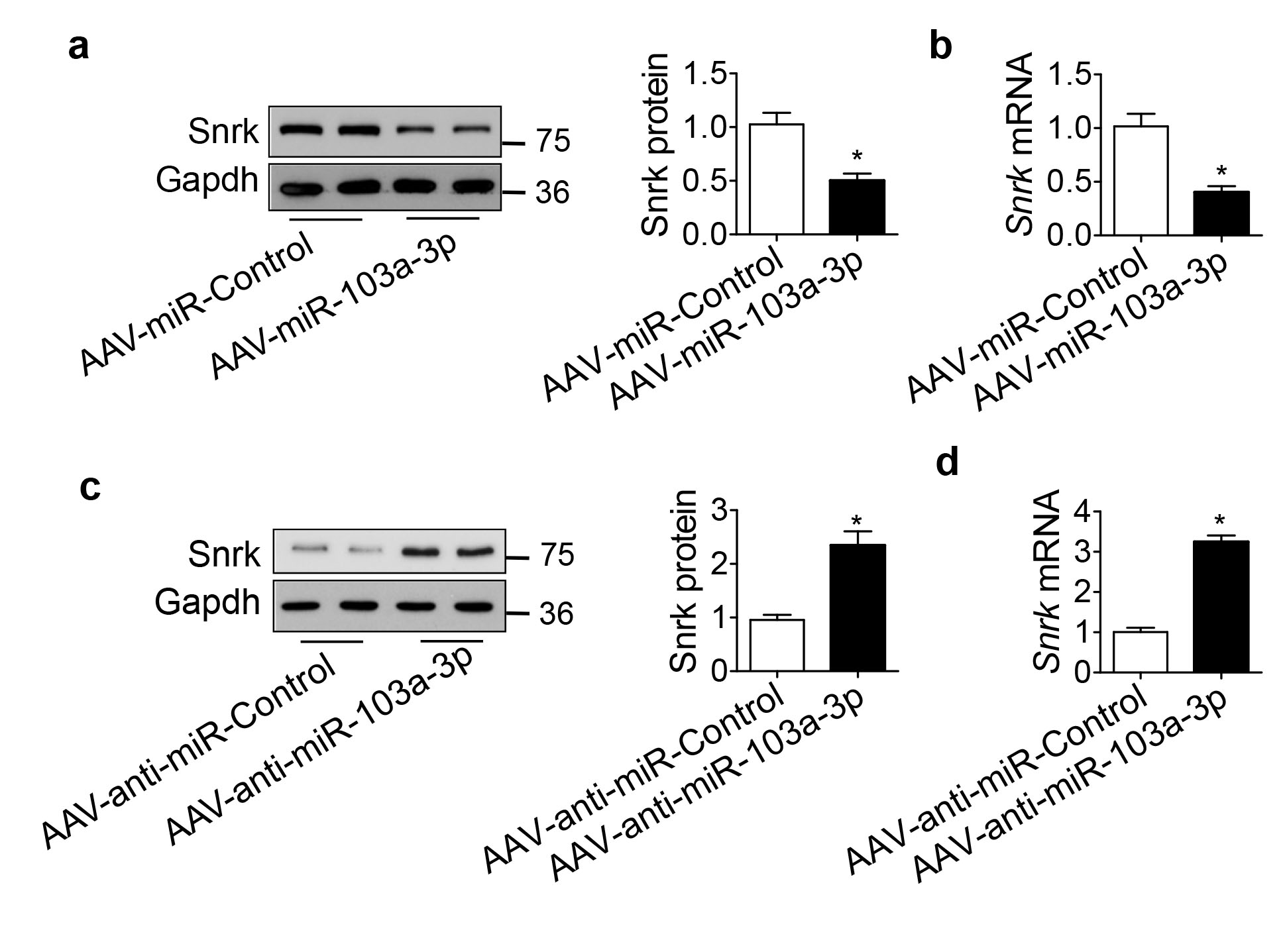
**

**Supplementary Figure 6. miR-103a-3p regulates Snrk expression *in vivo*. a** WT mice were transfected with AAV-miR-Control or AAV-miR-103a-3p. Renal Snrk protein levels were detected by Western blot. **b** Renal *Snrk* mRNA expression was analyzed by qRT-PCR. For all experiments, n = 6 per group; **p* < 0.05 relative to AAV-miR-Control. **c** and **d** WT mice were transfected with AAV-anti-miR-Control or AAV-anti-miR-103a-3p. Renal Snrk protein levels were detected by Western blot (**c**). **d** Renal *Snrk* mRNA expression was analyzed by qRT-PCR. For all experiments, n = 6 per group; **p* < 0.05 relative to AAV-anti-miR-Control. Statistical analysis was carried out with a Student’s two-tailed *t*-test. The corresponding source data are available in the Source Data file.

**
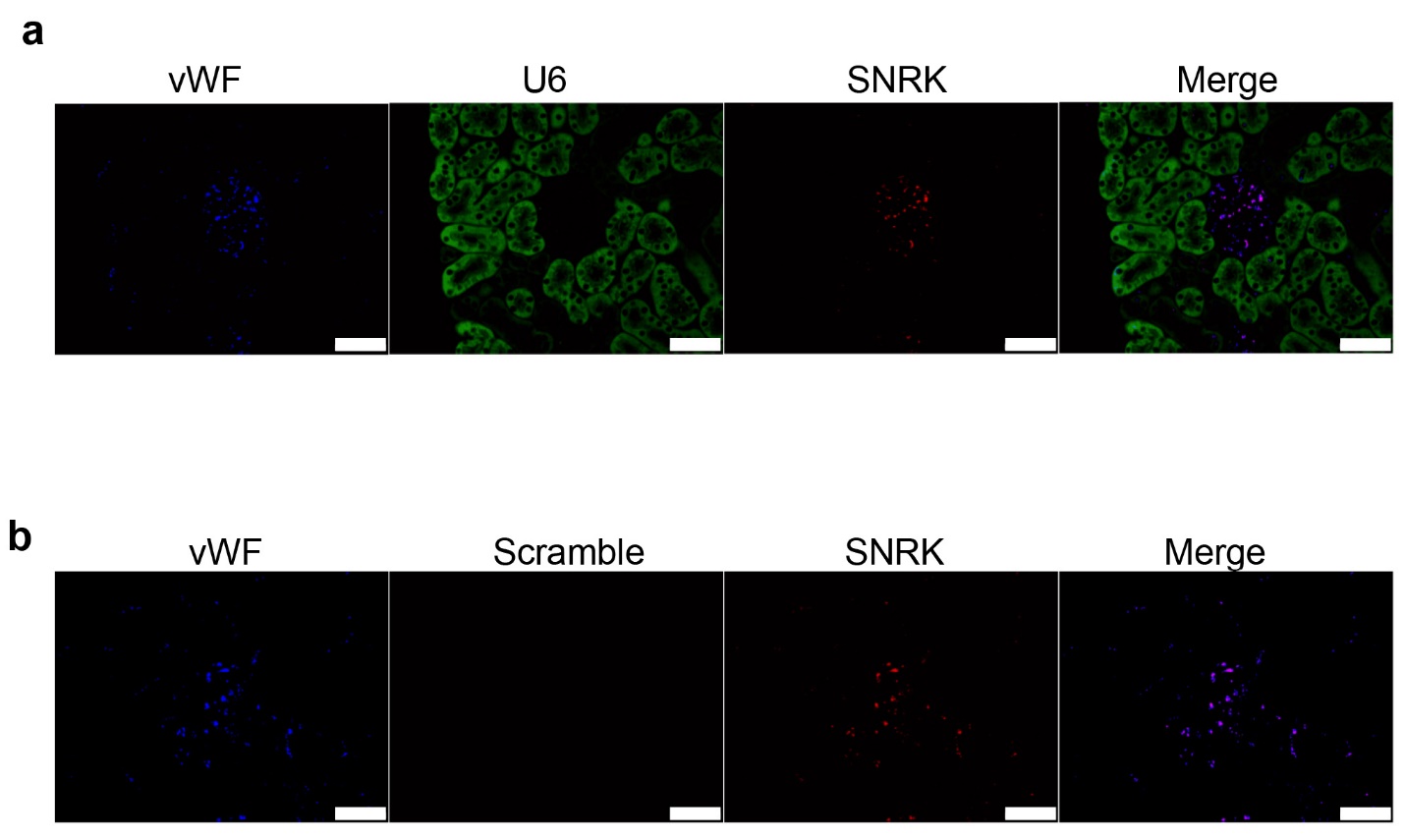
**

**Supplementary Figure 7. Positive and negative control for miRNAs staining.** Immunofluorescent co-staining of vWF (blue), SNRK (red), and U6 (green, positive control) (**a**) or Scramble (negative control) (**b**) in kidney tissues of healthy controls (scale bar=50 µm). The corresponding source data are available in the Source Data file.

**
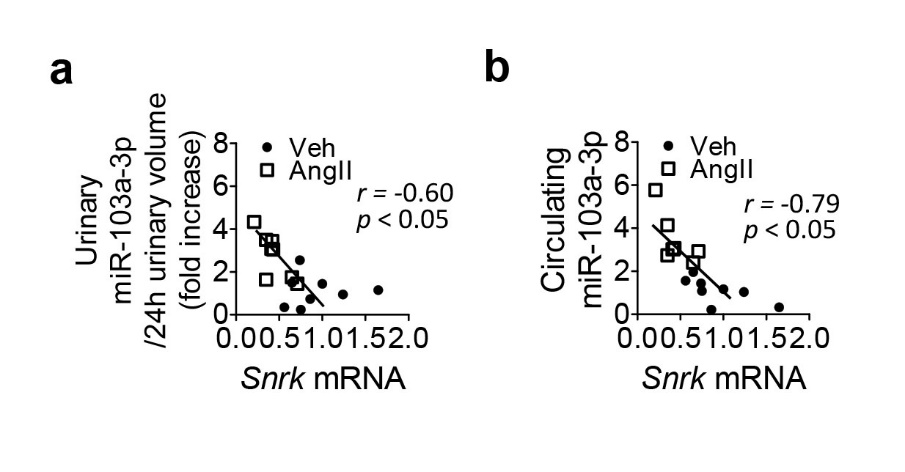
**

**Supplementary Figure 8. The level of Snrk expression is negatively related with miR-103a-3p in AngII-infused mice.** WT mice were infused with vehicle or AngII (1 mg kg^-1^ d^-1^) for four weeks. **a** Correlation between urinary miR-103a-3p and renal *Snrk* mRNA in in Veh- and AngII-infused mice. **b** Correlation between serum miR-103a-3p and renal *Snrk* mRNA in Veh- and AngII-infused mice. The corresponding source data are available in the Source Data file.

**
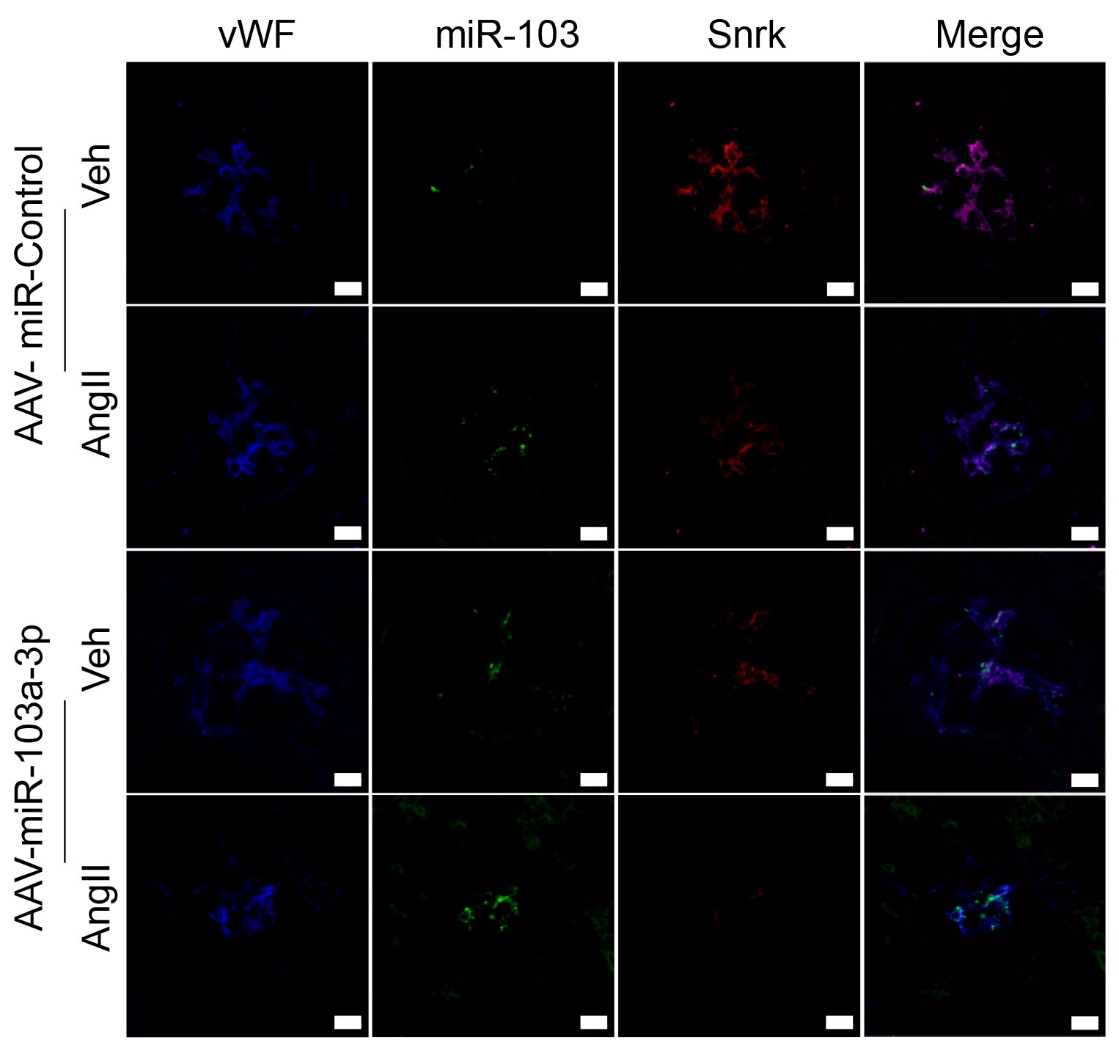
**

**Supplementary Figure 9. AAV-miR-103a-3p administration in mice causes the increase for miR-103a-3p in GnECs.** Mice injected with AAV-miR-103a-3p or AAV-miR-control were infused with Veh or AngII (1 mg kg^-1^ d^-1^) for 4 weeks. The kidney tissues were collected from these four group mice. IHC and FISH staining were performed with these kidney sections using vWF antibody (Blue), Snrk antibody (Red), and miR-103a-3p probe (Green) (scale bar=20 µm). The corresponding source data are available in the Source Data file.

**
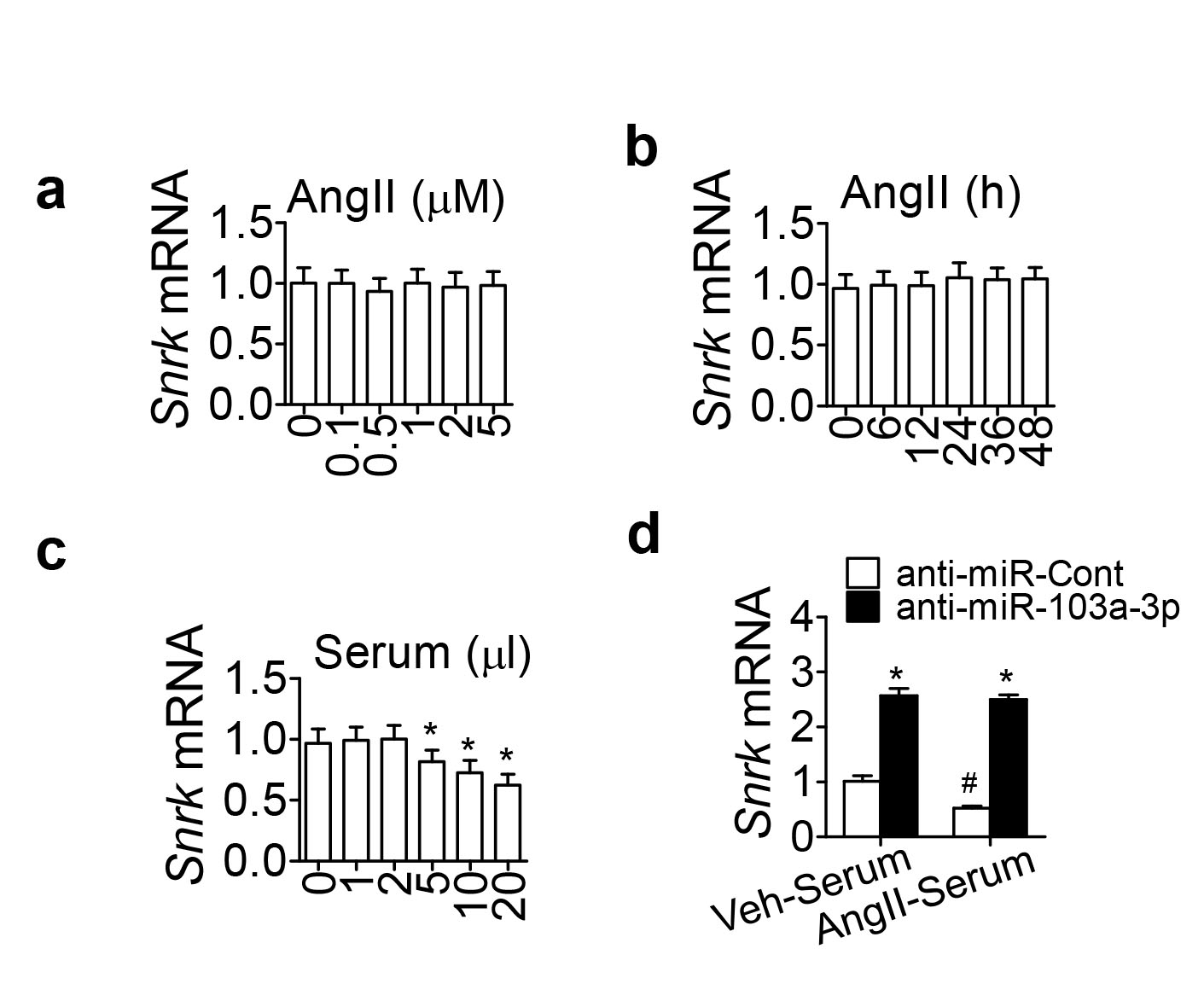
**

**Supplementary Figure 10. AngII-infused mouse serum reduces *Snrk* mRNA levels in cultured GnECs. a** *Snrk* mRNA levels in isolated primary GnECs from WT mice were analyzed by qRT-PCR after treatment with different doses of AngII. **b** *Snrk* mRNA levels in isolated GnECs from WT mice were analyzed after different incubation times with 1 µM AngII. **c** WT mice were infused with vehicle or AngII for 4 weeks, and serum was collected from these mice. GnECs were treated with different doses of AngII-treated mouse serum for 8 h, and *Snrk* levels were analyzed by qRT-PCR. n = 6; **p* < 0.05 relative to untreated controls. **d** The GnECs were pretreated with AAV-anti-miR-103a-3p or AAV-anti-miR-Control for 24 h and then incubated with serum (10 µl) from mice treated with Veh or AngII. *Snrk* mRNA levels were measured by qRT-PCR. n = 6 per group; **p* < 0.05 relative to anti-miR-Control; ^#^*p* < 0.05 relative to vehicle-treated mouse serum. Statistical analysis was carried out with a Student’s two-tailed *t*-test. The corresponding source data are available in the Source Data file.

**
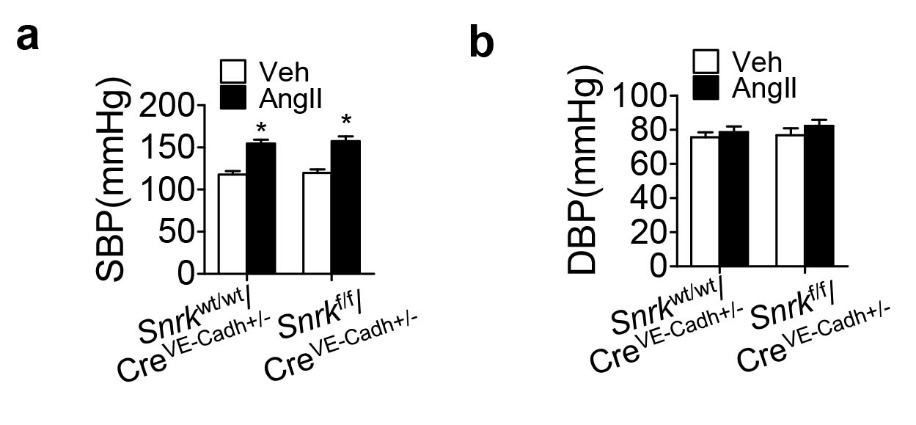
**

**Supplementary Figure 11. Endothelial *Snrk* deficiency does not affect the blood pressures. a** Systolic blood pressure (SBP) of *Snrk*^wt/wt^/Cre^VE-Cadh+/-^ and *Snrk*^f/f^/Cre^VE-Cadh+/-^ mice treated with Veh or AngII (n = 8, **p* < 0.05, vs. Vehicle). **b** Diastolic blood pressure (DBP) of *Snrk*^wt/wt^/Cre^VE-Cadh+/-^ and *Snrk*^f/f^/Cre^VE-Cadh+/-^ mice treated with Vehicle or AngII. Statistical analysis was carried out with a Student’s two-tailed *t*-test. The corresponding source data are available in the Source Data file.

**
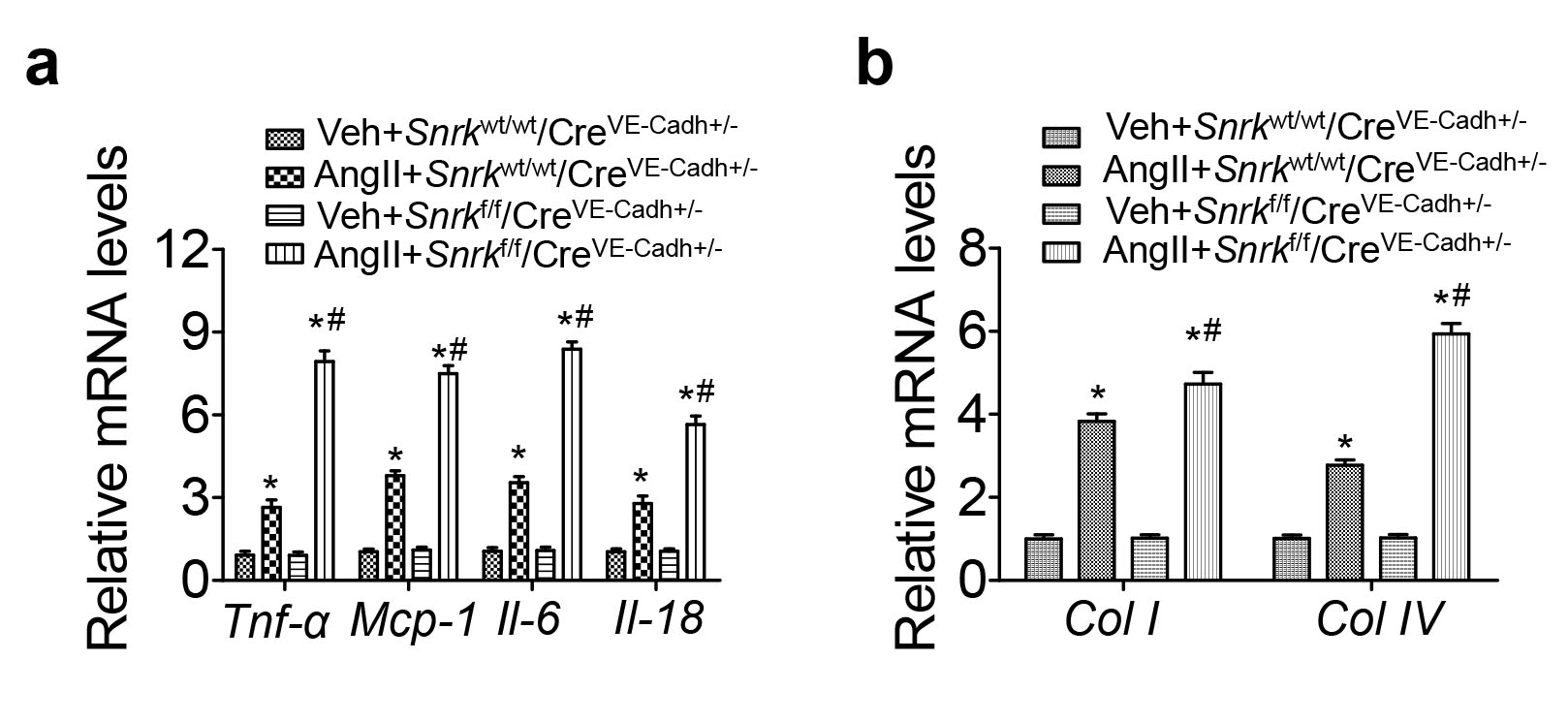
**

**Supplementary Figure 12. Deletion of endothelial *Snrk* exacerbates AngII-induced inflammatory response.** Eight-week-old male WT (*Snrk*^wt/wt^/Cre^VE-Cadh+/-^) and *Snrk* endothelial specific knockout (*Snrk*^f/f^/Cre^VE-Cadh+/-^) mice were infused with vehicle or AngII for 4 weeks. **a** The mRNA levels of *Mcp-1*, *Tnf-α*, *Il-6*, and *Il-18* in the kidneys of vehicle- and AngII-treated WT and *Snrk*^f/f^/Cre^VE-Cadh+/-^ mice. **b** The mRNA levels of *Col I* and *Col IV* in kidneys were showed. For all experiments, n = 8 per group; **p* < 0.05 relative to vehicle control; ^#^*p* < 0.05 relative to WT mice/AngII. Statistical analysis was carried out with a Student’s two-tailed *t*-test. The corresponding source data are available in the Source Data file.

**
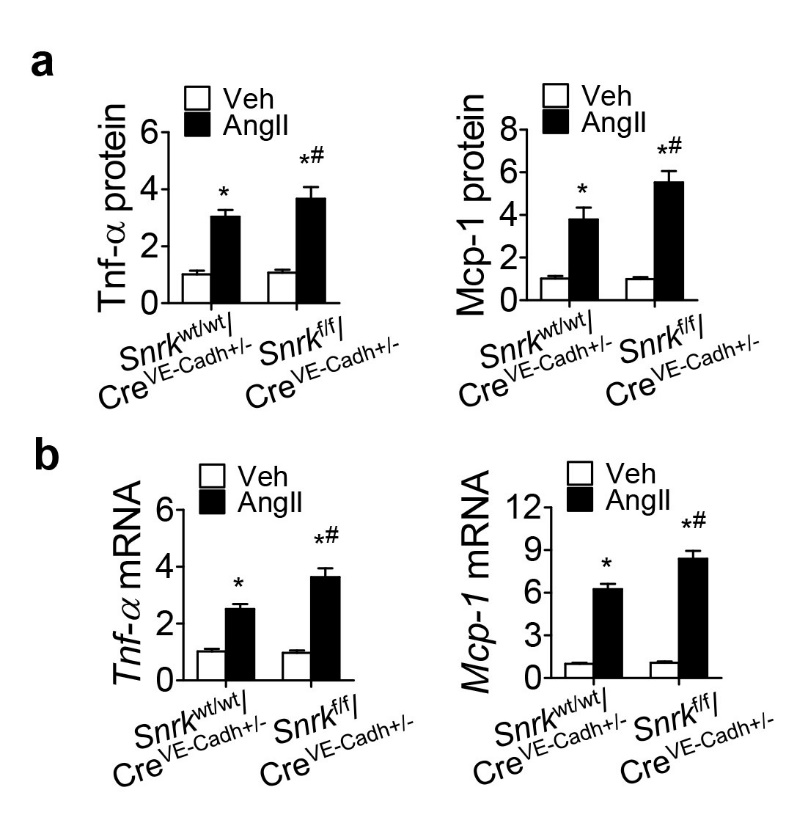
**

**Supplementary Figure 13. *Snrk* deficiency increases mRNA levels of proinflammatory factors in AngII-treated GnECs. a** Tnf-α and Mcp-1 protein levels were quantified by densitometric analysis of the Western bots. n = 6 per group; **p* < 0.05 relative to vehicle control; ^#^*p* < 0.05 relative to WT mice/AngII. **b** GnECs isolated from *Snrk*^wt/wt^/Cre^VE-Cadh+/-^ and *Snrk*^f/f^/Cre^VE-Cadh+/-^ mice were treated with/without AngII (1 µM) for 24 h. *Tnf-α* and *Mcp-1* mRNA levels were analyzed by qRT-PCR. n = 8 per group; **p* < 0.05 relative to vehicle control; ^#^*p* < 0.05 relative to WT mice/AngII. Statistical analysis was carried out with a Student’s two-tailed *t*-test. The corresponding source data are available in the Source Data file.

**
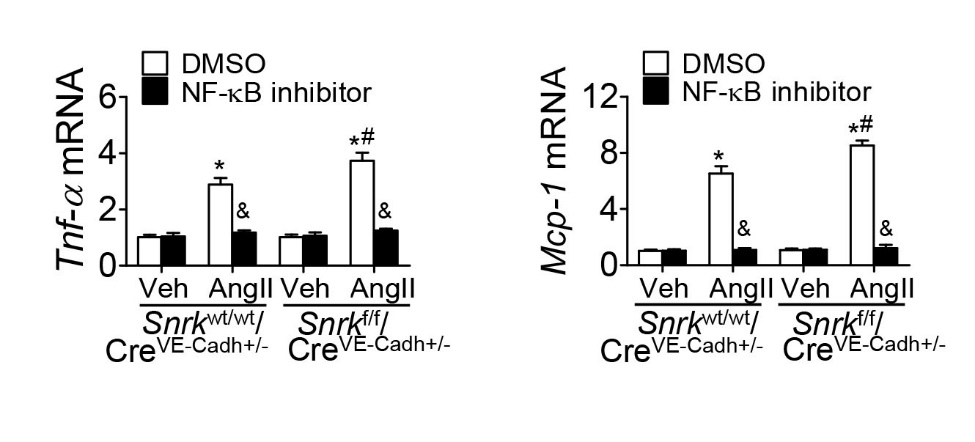
**

**Supplementary Figure 14. NF-κB inhibitor suppresses AngII-enhanced proinflammatory factors in *Snrk*- deficient GnECs.** The GnECs from *Snrk*^wt/wt^/Cre^VE-Cadh+/-^ and *Snrk*^f/f^/Cre^VE-Cadh+/-^ mice were pretreated with NF-κB inhibitor (1 µg mL^-1^, sc-3060, Santa Cruz Biotechnology) for 2 h, followed by treatment with AngII for 24 h. *Tnf-α* and *Mcp-1* mRNA levels were assayed by qRT-PCR. n = 6, **p* < 0.05 relative to vehicle control; ^#^*p* < 0.05 relative to GnECs from WT mice/AngII; ^&^*p* < 0.05 relative to DMSO control/AngII. Statistical analysis was carried out with a Student’s two-tailed *t*-test. The corresponding source data are available in the Source Data file.

**
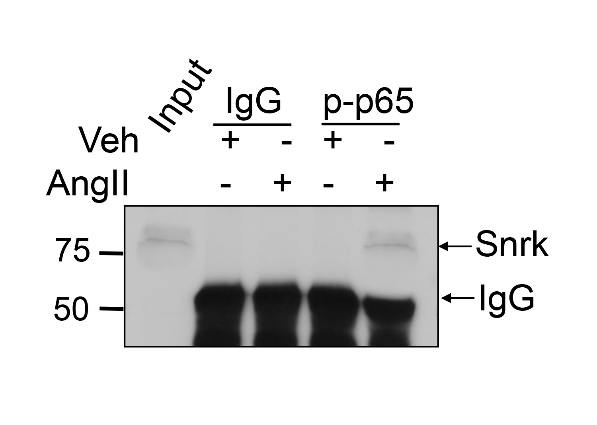
**

**Supplementary Figure 15. The interaction between p-p65 and Snrk.** The GnECs were treated with AngII or vehicle for 24 h. The interaction of Snrk and p-p65 were analyzed using immunoprecipitation and Western blotting (input: total cell lysates; IgG: immunoprecipitated with IgG). The corresponding source data are available in the Source Data file.

**
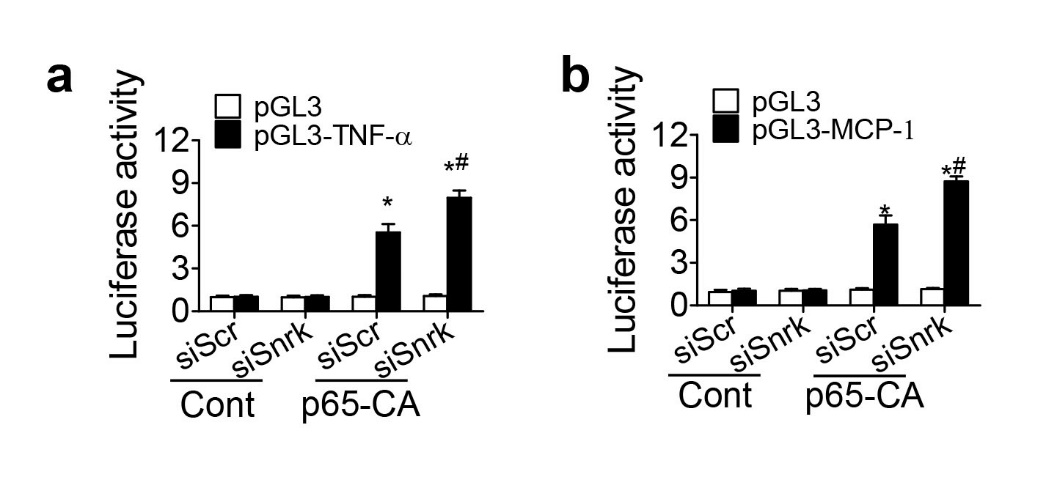
**

**Supplementary Figure 16. *Snrk* deficiency in GnECs enhances Tnf-α and Mcp-1 expression by promoting p65 activity. a** *Snrk* knockdown increased p65-CA-induced luciferase activity of *Tnf-α* promoter. n = 5, **p* < 0.05 relative to pGL3; ^#^*p* < 0.05 relative to si*Scr*. **b** *Snrk* knockdown increased p65-CA-induced luciferase activity of *Mcp-1* promoter. n = 5, **p* < 0.05 relative to pGL3; ^#^*p* < 0.05 relative to si*Scr*. Statistical analysis was carried out with a Student’s two-tailed *t*-test. The corresponding source data are available in the Source Data file.

**
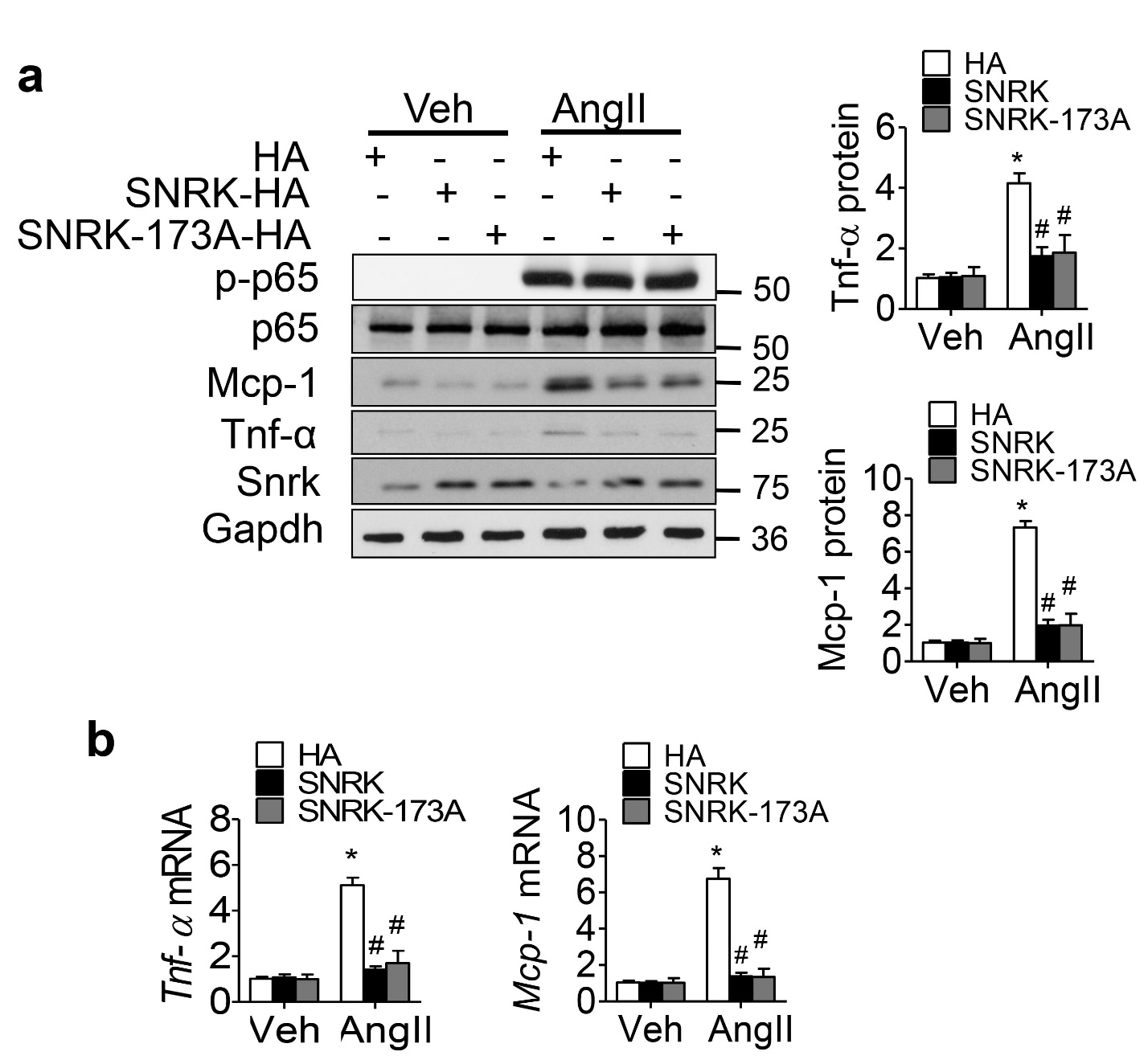
**

**Supplementary Figure 17. SNRK regulates p65 transcriptional activity independently of SNRK kinase activity.** GnECs were transfected with hemagglutinin (HA)- tagged wild-type SNRK (SNRK-HA) or SNRK-T173A mutant (SNRK-T173A) for 24 h, and then treated with AngII (1 µM) or vehicle for 24 h. **a** Western blot analysis of Mcp-1, Snrk, Tnf-α, p-p65, p65 and Gapdh (n = 5, **p* < 0.05 vs. Vehicle; #*p* < 0.05 vs. HA). **b** *Mcp-1* and *Tnf-α* mRNA levels were detected by qRT-PCR (n = 5, **p* < 0.05 vs. Vehicle; #*p* < 0.05 vs. HA). Statistical analysis was carried out with a Student’s two-tailed *t*-test. The corresponding source data are available in the Source Data file.

**
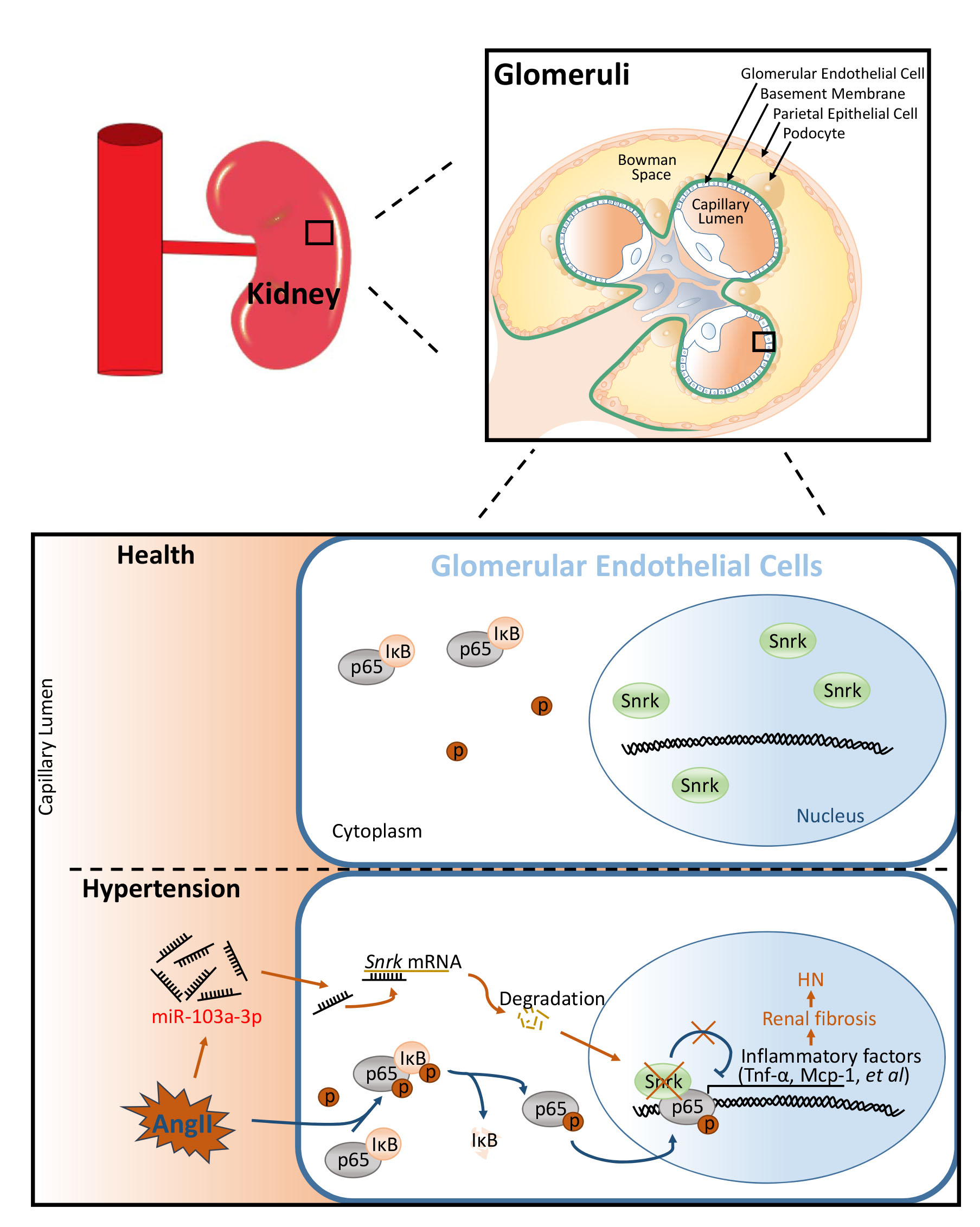
**

**Supplementary Figure 18. The proposed signal pathway for circulating miR-103a-3p/Snrk contributing to AngII-induced renal injury.** AngII-induced increase in circulating miR-103a-3p reduces glomerular endothelial Snrk. Snrk binds to phosphorylated p65 and suppresses its transcriptional abilities, suppressing AngII-induced renal damage. The blue arrows indicate the effect of AngII on glomerular endothelial cells. The orange arrows indicate the effects of AngII on kidneys.

**Supplementary Figure 19. Uncropped immunoblots of the different figures.**

**
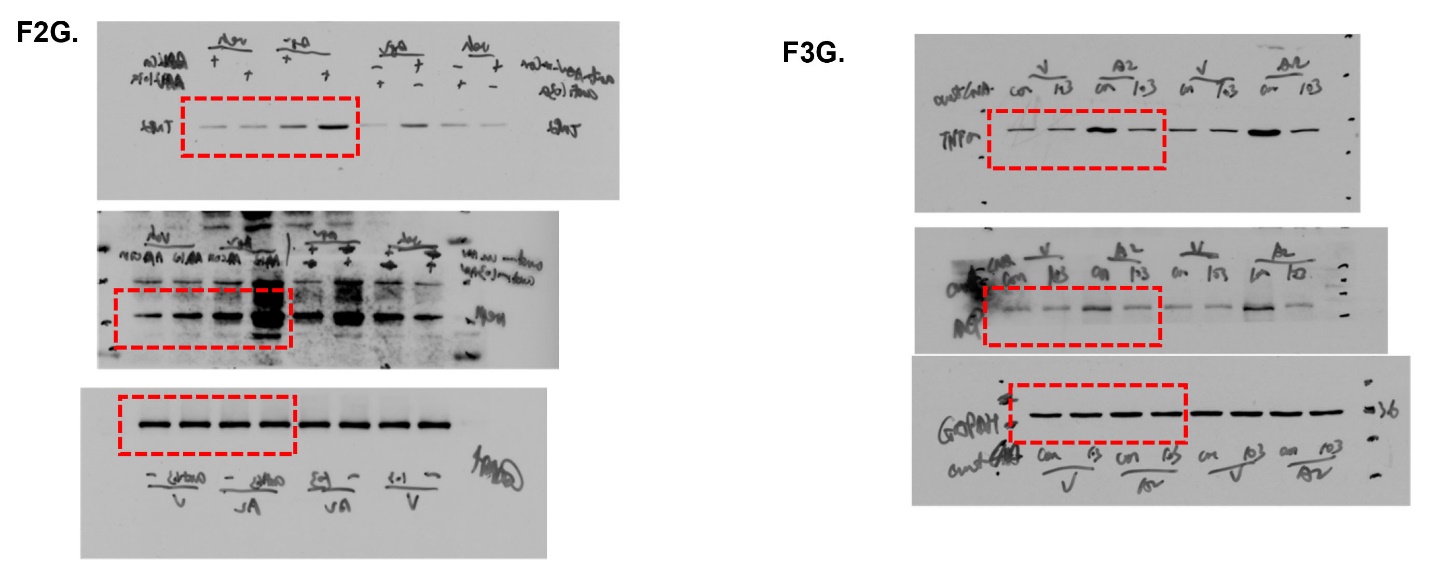
**

**
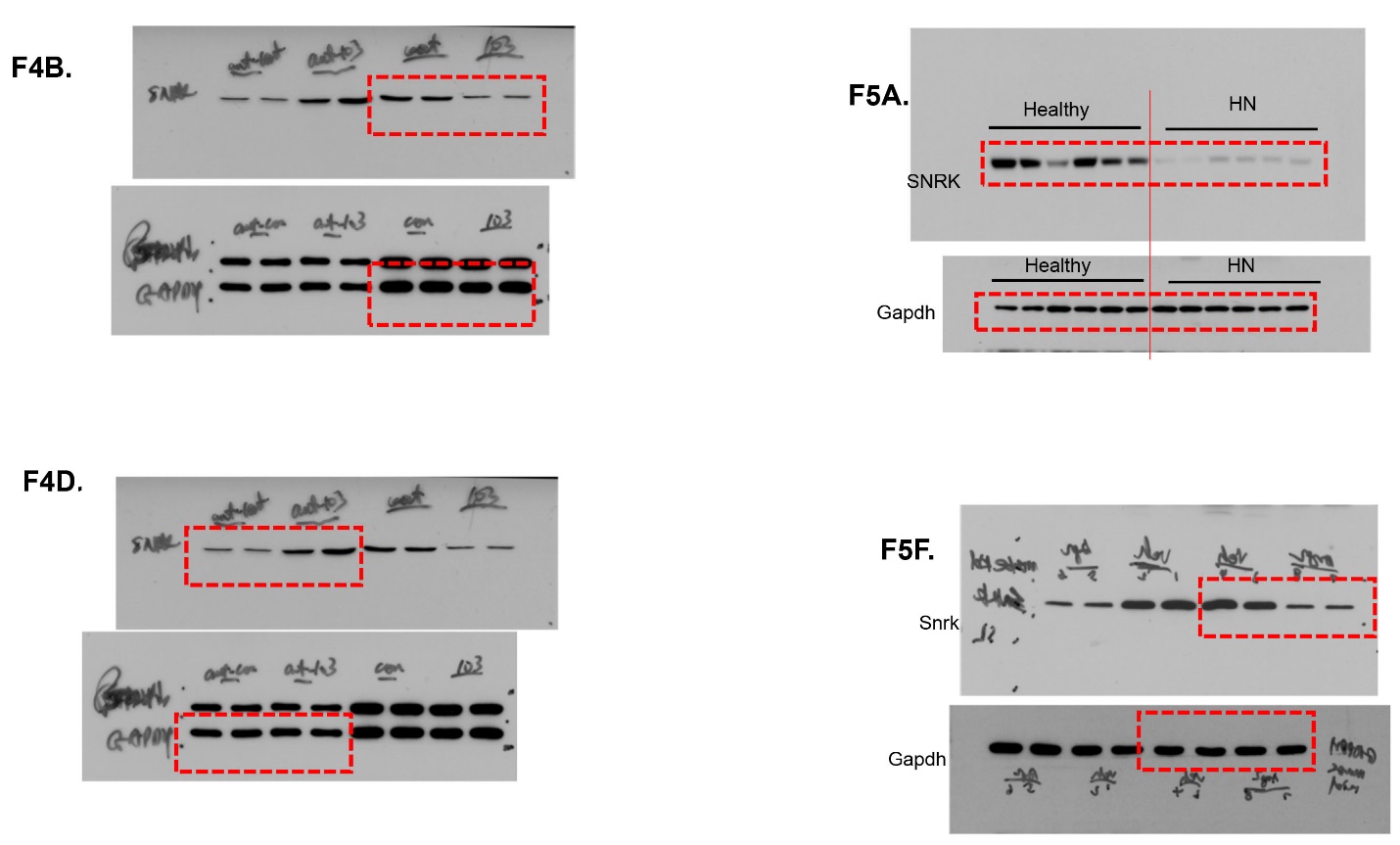

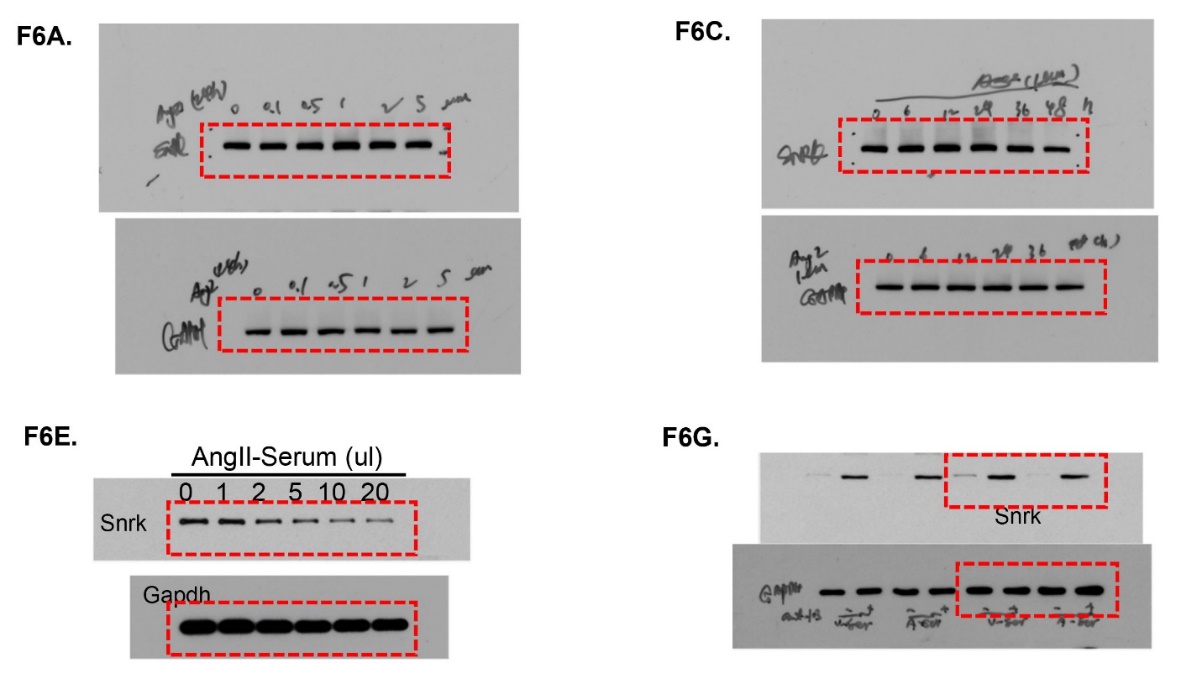
**

**
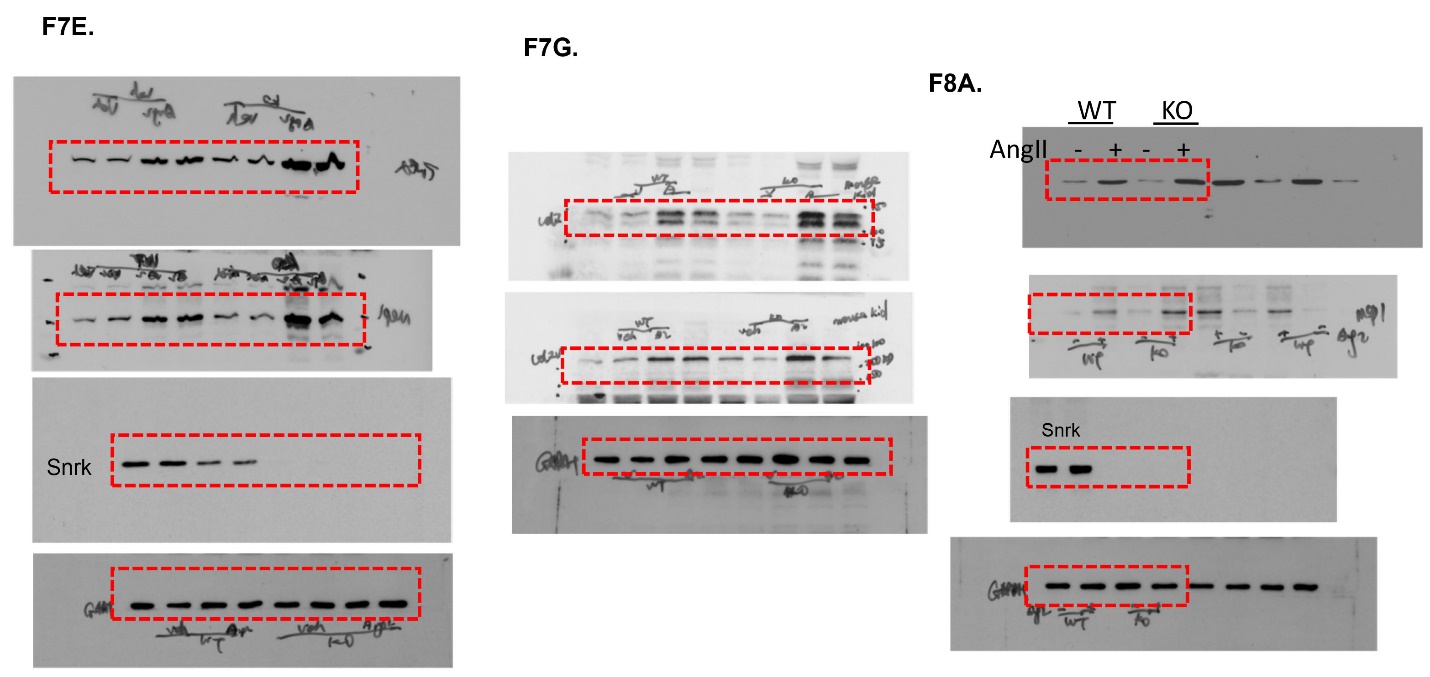
**

**
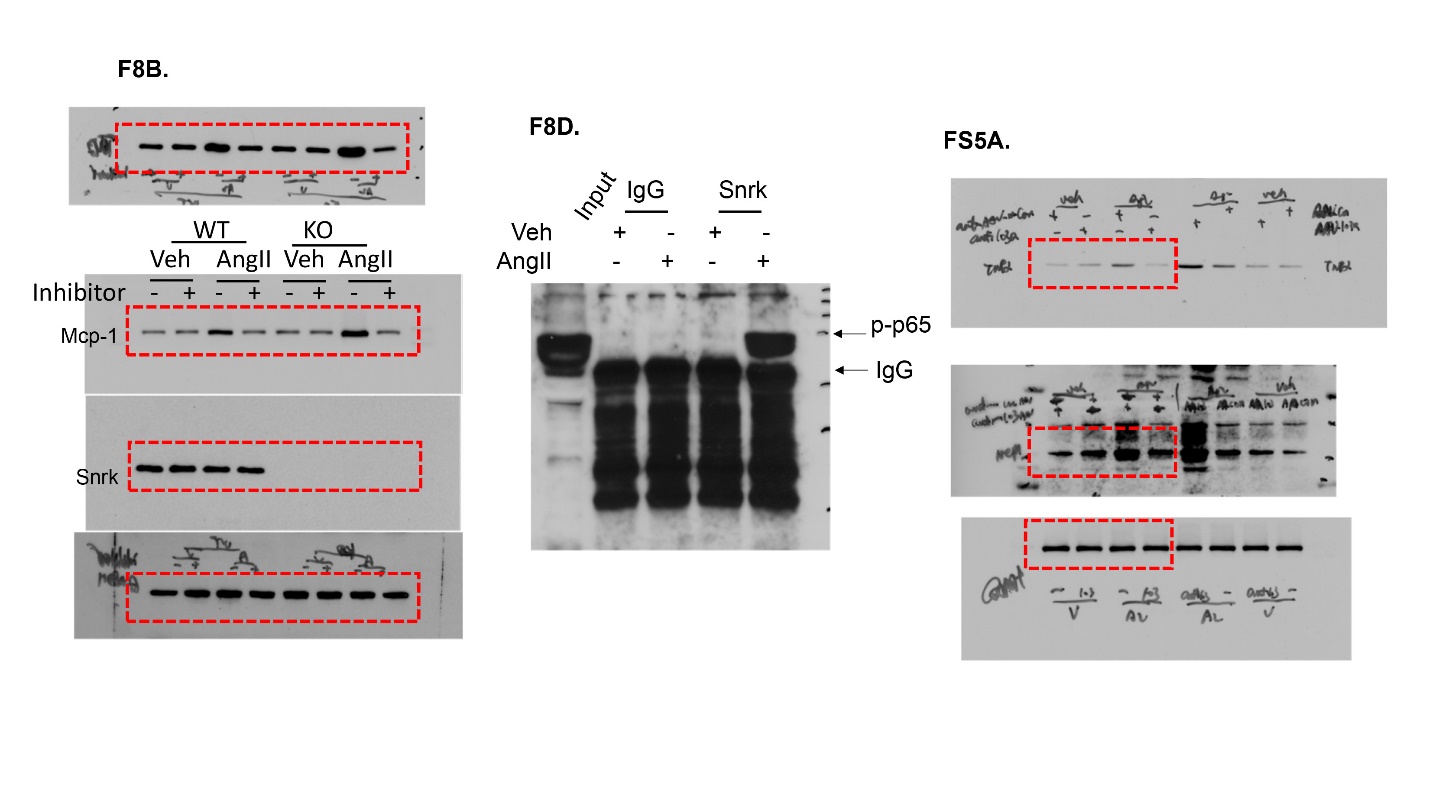
**

**
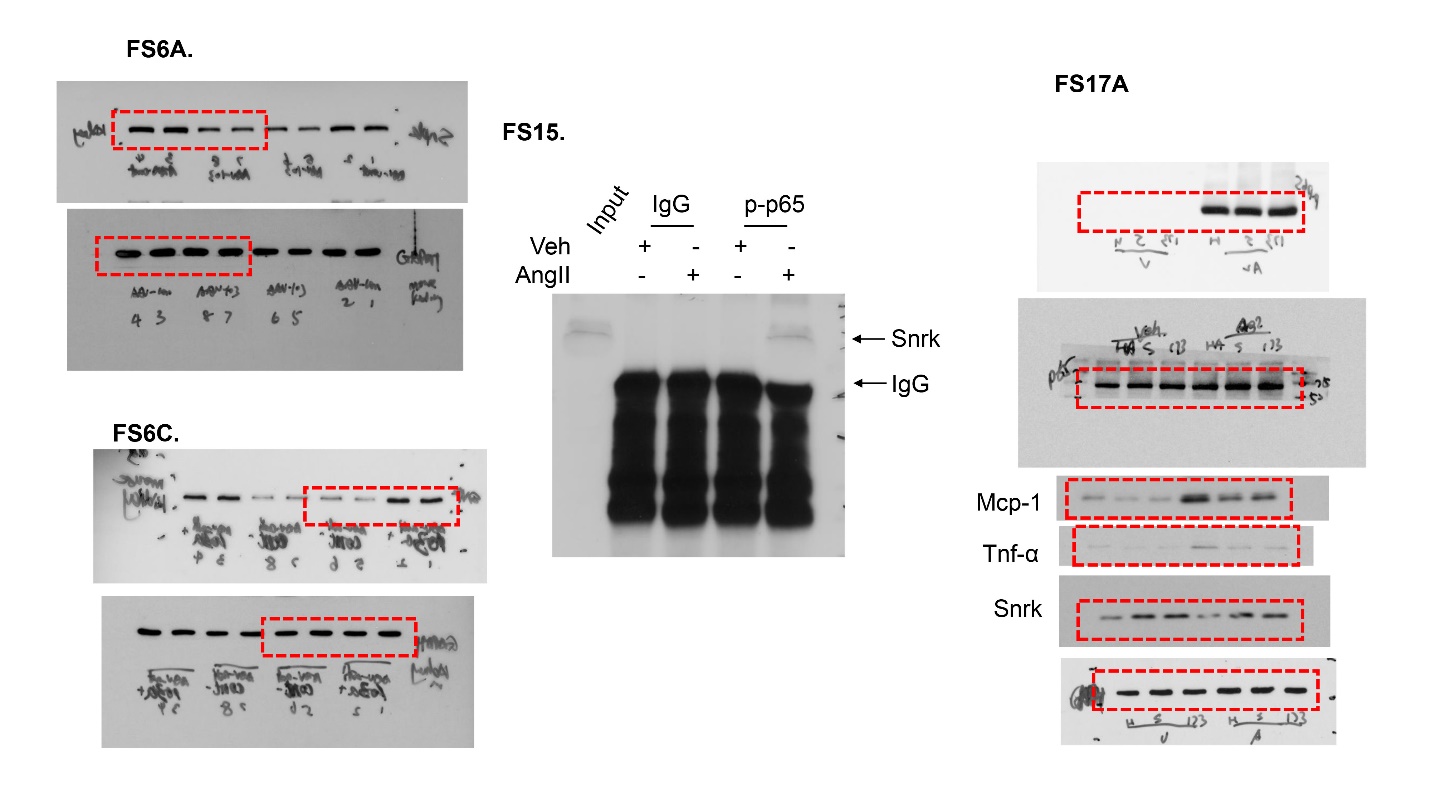
**

**Supplementary Table 1. Basic clinical information for cohorts of healthy and patients with no-medically treated HN.**

| **Characteristic** | **Healthy** | **No-medically treated HN** |
| --- | --- | --- |
| Number | 18 | 31 |
| Age | 41.9±10.4 | 42.6±7.4 |
| Gender (M/F) | 8/10 | 16/15 |
| BMI（kg/m^2^） | 23.17±3.57 | 23.45±2.92 |
| SBP（mmHg） | 120.1±9.72 | 149.7±12.57* |
| DBP（mmHg） | 75.7±8.33 | 95.3±5.82* |
| FPG（mmol/L） | 5.56±1.32 | 5.45±1.64 |
| TG（mmol/L） | 1.17±0.22 | 1.25±0.25 |
| TC（mmol/L） | 4.12±0.72 | 4.27±0.65 |
| ACR (mg/g) | 27.31±9.32 | 641.84±314.30* |
| eGFR (mL/min) | 89.23±3.62 | 49.26±2.68* |

Note: Body mass index (BMI); systolic blood pressure (SBP); diatolic blood pressure (DBP); fasting plasma glucose (FPG); triglyceride (TG); total cholesterol (TC); albumin-to-creatinine ratio (ACR) estimated glomerular filtration rate (eGFR). **p* < 0.05 vs. Healthy.

**Supplementary Table 2. The primers were used for plasmid generation.**

| **Plasmid** | **Forward** | **Reverse** |
| --- | --- | --- |
| pCMV-SNRK-HA | GTCGGAATTCGGGCAGGATTTAAGCGAG | ATGCCTCGAGGATGACATGGCAACAGCT |
| pCMV-SNRK-T173A-HA | GAAGCTCACTGCAAGCTGTGGATCTCTTG | AGCTTGCAGTGAGCTTCTTCCCTGGTTG |
| pMIR-167 | GGGCAAGCTTCCGGCTCACTTCACTGTTCCATTTG | CGCCGAGCTCCCACATCACAGAAGGTC AGGACAGGA |
| pMIR-1994 | GGGCAAGCTTGCATTGCTGTGGTCACTGTTTCTTC | CGCCGAGCTCGGTTTTTCGCTCCCTCCCTTTTATGT |
| pMIR-167-Δ | TGTGCTAGACACTTTTCTTTCCCAGCCG | GTCTAGCACAAAGACCATGCATTGTCAC |
| pMIR-1994-Δ | AGCAGTGTTGACCATTTATAGCTGTAT | CTATAAATGGTCAACACTGCTGATCCT |
| pGL-MCP-1 | CTCAAAGGTGCTGCAGAGTTACTT | TGCATAGTGGTGGAGGAAGA |
| pGL-TNF-α | ATGCGGTACCGAGCTCCTGGGAGATATGGC | ATGCCTCGAGCGCCTGCCACGATCAGGAAG |
| AAV- miR-103a-3p | GATCCAGCAGCATTGTACAGGGCTATGATTCAAGAGATCATAGCCCTGTACAATGCTGCTCCGC | GGCCGCGGAGCAGCATTGTACAGGGCTATGATCTCTTGAATCATAGCCCTGTACAATGCTGCTG |
| AAV-anti- miR-103a-3p | GATCCTCATAGCCCTGTACAATGCTGCTTTCAAGAGAAGCAGCATTGTACAGGGCTATGACCGC | GGCCGCGGTCATAGCCCTGTACAATGCTGCTTCTCTTGAAAGCAGCATTGTACAGGGCTATGAG |
| AAV-miR-Random | GATCCTTTGTACTACACAAAAGTACTGTTCAAGAGACAGTACTTTTGTGTAGTACAAACCGC | GGCCGCGGTTTGTACTACACAAAAGTACTGTCTCTTGAACAGTACTTTTGTGTAGTACAAAG |

**Supplementary Table 3. The primers used for mRNA quantification in real time PCR assays.**

|  | Gene | Forward | Reverse |
| --- | --- | --- | --- |
| human | SNRK | CCCGAGCAATATCAAGGCCCAGTT | TCGCGTGGGACAAAGGAGTGG |
| mouse | Snrk | CGCGAGCTGTCAGGGGAA | AACCAAAGAGTAAAG |
| mouse | Mcp-1 | GCATCCACGTGTTGGCTC | CTCCAGCCTACTCATTGGGATCA |
| mouse | *Tnf-α* | CACGTCGTAGCAAACCACCAAGTGGA | TGGGAGTAGACAAGGTACAACCC |
| mouse | *Il-6* | TCTATACCACTTCACAAGTCGGA | GAATTGCCATTGCACAACTCTTT |
| mouse | *Il-18* | GTGAACCCCAGACCAGACTG | CCTGGAACACGTTTCTGAAAGA |
| mouse | *Col I* | GCTCCTCTTAGGGGCCACT | CCACGTCTCACCATTGGGG |
| mouse | *Col IV* | CTGGCACAAAAGGGACGAG | ACGTGGCCGAGAATTTCACC |
| human | *GAPDH* | GCTGAGACTGGGCCAATT | AGCAAAATCAAAACTTGATT |
| mouse | *Gapdh* | CAGTCCAGTCAATTCAACCA | CGGCCATCCCTAAGCTCC |

Note: Sucrose non-fermenting 1-related kinase (SNRK); monocyte chemoattractant protein-1 (Mcp-1); tumor necrosis factor-α (Tnf-α); interleukin-6 (Il-6); interleukin-18 (Il-18); Collagen I (Col I); Collagen IV (Col IV).
