# Supplementary material for: Circulating miR-103a-3p contributes to angiotensin II-induced renal inflammation and fibrosis via a SNRK/NF-κB/p65 regulatory axis
Source: Nat Commun. 2019 May 13;10:2145. doi: 10.1038/s41467-019-10116-0 (PMC6513984; doi:10.1038/s41467-019-10116-0)
Supplement: Supplementary file 2 — Reporting Summary [file 41467_2019_10116_MOESM2_ESM.pdf]

nature research

Corresponding author(s): Ping Song

Last updated by author(s): Apr 13, 2019

Reporting Summary

Nature Research wishes to improve the reproducibility of the work that we publish. This form provides structure for consistency and transparency in reporting. For further information on Nature Research policies, see Authors & Referees and the Editorial Policy Checklist.

Statistics

For all statistical analyses, confirm that the following items are present in the figure legend, table legend, main text, or Methods section.

n/a	Confirmed	
☐	☒	The exact sample size (*n*) for each experimental group/condition, given as a discrete number and unit of measurement
☐	☒	A statement on whether measurements were taken from distinct samples or whether the same sample was measured repeatedly
☐	☒	The statistical test(s) used AND whether they are one- or two-sided<br>*Only common tests should be described solely by name; describe more complex techniques in the Methods section.*
☒	☐	A description of all covariates tested
☒	☐	A description of any assumptions or corrections, such as tests of normality and adjustment for multiple comparisons
☐	☒	A full description of the statistical parameters including central tendency (e.g. means) or other basic estimates (e.g. regression coefficient) AND variation (e.g. standard deviation) or associated estimates of uncertainty (e.g. confidence intervals)
☒	☐	For null hypothesis testing, the test statistic (e.g. *F*, *t*, *r*) with confidence intervals, effect sizes, degrees of freedom and *P* value noted<br>*Give P values as exact values whenever suitable.*
☒	☐	For Bayesian analysis, information on the choice of priors and Markov chain Monte Carlo settings
☒	☐	For hierarchical and complex designs, identification of the appropriate level for tests and full reporting of outcomes
☒	☐	Estimates of effect sizes (e.g. Cohen's *d*, Pearson's *r*), indicating how they were calculated

Our web collection on statistics for biologists contains articles on many of the points above.

Software and code

Policy information about availability of computer code

Data collection	SPSS version 20, GraphPad Prism 5
Data analysis	SPSS version 20, GraphPad Prism 5

For manuscripts utilizing custom algorithms or software that are central to the research but not yet described in published literature, software must be made available to editors/reviewers. We strongly encourage code deposition in a community repository (e.g. GitHub). See the Nature Research guidelines for submitting code & software for further information.

Data

Policy information about availability of data

All manuscripts must include a data availability statement. This statement should provide the following information, where applicable:

- Accession codes, unique identifiers, or web links for publicly available datasets
- A list of figures that have associated raw data
- A description of any restrictions on data availability

All data are available from the corresponding author upon reasonable request. The source data underlying all main and supplementary figures are available as a Source Data file.

Field-specific reporting

Please select the one below that is the best fit for your research. If you are not sure, read the appropriate sections before making your selection.

☒ Life sciences ☐ Behavioural & social sciences ☐ Ecological, evolutionary & environmental sciences

October 2018

Life sciences study design

All studies must disclose on these points even when the disclosure is negative.

Sample size	We collected urine and serum samples from 60 newly diagnosed hypertensive patients (SBP > 130 mm Hg) and 18 individuals with normal blood pressure (SBP < 130 mm Hg). 31 patients with hypertensive nephropathy (HN, n=31) out of 60 patients received no medical treatment while 29 patients with renal dysfunctions were treated with either Diovan (β-blockers) (n= 13) or Monopril (ACEi) (n = 16) for 2-3 weeks.<br>For LNA-anti-miRNA experiments, one day after mini-pump implantation, mice (n =8 mice per group) received 2 intravenous injections of 25 mg/kg of LNA inhibitors (LNA-anti-miR-Control or LNA-anti-miR-103a-3p) per week for three weeks.
Data exclusions	Animals were excluded if mice were died.
Replication	Repeat experimental measurements were taken, all experiments were replicated as specified in figure legends.
Randomization	Animals were randomly assigned to the different study groups.
Blinding	Blinding was conducted for animal and human data collection.

Reporting for specific materials, systems and methods

We require information from authors about some types of materials, experimental systems and methods used in many studies. Here, indicate whether each material, system or method listed is relevant to your study. If you are not sure if a list item applies to your research, read the appropriate section before selecting a response.

Materials & experimental systems

n/a	Involved in the study	
☐	☒	Antibodies
☐	☒	Eukaryotic cell lines
☒	☐	Palaeontology
☐	☒	Animals and other organisms
☐	☒	Human research participants
☒	☐	Clinical data

Methods

n/a	Involved in the study	
☒	☐	ChIP-seq
☒	☐	Flow cytometry
☒	☐	MRI-based neuroimaging

Antibodies

Antibodies used	WB: Primary antibodies and dilutions used were as follows: SNRK (1:1000; ab96762, Abcam), MCP-1 (1:1000; ab7202, Abcam), TNF-α (1;1000; 3703, Cell Signal Technology), Col I (1:1000; 84336, Cell Signal Technology), and Col IV (1:200; ab6586, Abcam).<br>Immunofluorescence: primary antibodies (anti-vWF, ab11713 from Abcam, 1:100; anti-SNRK from Abcam, 1:100)<br>Immunohistochemistry: Primary antibodies and dilutions used were as follows: Col IV (1:200; ab6586, Abcam), SNRK (1:500; ab96762, Abcam).
Validation	All the antibodies were validated by the company.

Eukaryotic cell lines

Policy information about cell lines

Cell line source(s)	HEK293 cell line was purchased from ATCC.
Authentication	The cell line was purchased from ATCC and was authenticated by ATCC.
Mycoplasma contamination	The cell line tested negative.
Commonly misidentified lines (See ICLAC register)	There is no cell lines used appear in the ICLAC register.

Animals and other organisms

Policy information about studies involving animals; ARRIVE guidelines recommended for reporting animal research

Laboratory animals	All animal (mouse with C57 BL/6J background) experimental protocols were approved by the Institutional Animal Care and Use Committee at Georgia State University and conformed to NIH guidelines.

Wild animals	The study did not involve wild animals.
Field-collected samples	N/A
Ethics oversight	Georgia State University IACUC approved the animal study protocol.

Note that full information on the approval of the study protocol must also be provided in the manuscript.

Human research participants

Policy information about studies involving human research participants

Population characteristics	We collected urine and serum samples from 60 newly diagnosed hypertensive patients (SBP > 130 mm Hg) and 18 individuals with normal blood pressure (SBP < 130 mm Hg). 31 patients with hypertensive nephropathy (HN, n=31) out of 60 patients received no medical treatment while 29 patients with renal dysfunctions were treated with either Diovan (β-blockers) (n= 13) or Monopril (ACEi) (n = 16) for 2-3 weeks.
Recruitment	The human subject studies were approved and performed according to the standards established by the Ethics Committee on Human Subject Research at Tianjin Medical University. We have complied with all relevant ethical regulations. Written informed consent was received from all human participants prior to inclusion in the study.
Ethics oversight	The Ethics Committee on Human Subject Research at Tianjin Medical University approved the human subject studies.

Note that full information on the approval of the study protocol must also be provided in the manuscript.
